# Myofascial chain sequencing and performance adaptations in youth football players: an exploratory knowledge graph-supported analysis

**DOI:** 10.1186/s13102-026-02066-0

**Published:** 2026-09-08

**Authors:** İnci Kesilmiş, Aybike Şimşek, Aleyna Işik, Mehmet Şimşek

**Affiliations:** 1 Department of Coaching Education, Faculty of Sports Sciences, Mersin University, Mersin, 33343 Turkey; 2 Exercise and Sports Sciences Application and Research Center, Mersin University, Mersin, 33343 Turkey; 3 Department of Computer Engineering, Faculty of Engineering and Architecture, Sinop University, Sinop, 57000 Türkiye; 4 Department of Physical Education and Sports, Graduate School of Educational Sciences, Mersin University, Mersin, 33343 Turkey

**Keywords:** Myofascial chain sequencing, Posterior chain, Balance, Countermovement jump, Knowledge graph

## Abstract

**Background:**

This study investigated the effects of myofascial chain-focused training on countermovement jump (CMJ) performance and dynamic balance in a small, specific cohort of youth football players, while also examining how performance changed across the intervention with different chain sequences.

**Methods:**

Forty licensed adolescent male football players (U15–U16) participated in a 12-week intervention program consisting of training protocols based on distinct myofascial chain sequences (posterior-chain-first vs. spiral-chain-first). Performance assessments were conducted at four time points (pre-test, mid-test, post-test, and follow-up) using the CMJ and Y-Balance Test (YBT). The resulting data were analyzed through both conventional statistical approaches and a knowledge graph-based framework designed to model phase-dependent adaptation dynamics.

**Results:**

ANOVA revealed a significant main effect of time for all variables (CMJ: F₁.₅₁₅,₅₇.₅₈₁ = 69.15, *p* < .001, ηp² = 0.645; YBT-R: F₁.₀₃₃,₃₉.₂₆₈ = 17.12, *p* < .001, ηp² = 0.311; YBT-L: F₁.₀₅₂,₃₉.₉₇₂ = 11.45, *p* < .001, ηp² = 0.232). However, the Time × Group interaction was not significant for any variable (*p* > .05 for all), indicating similar overall improvement trajectories between sequences. Pairwise comparisons showed that CMJ improved progressively across all time points (pre-to-follow-up mean difference = 7.57 cm, *p* < .001). Descriptive knowledge graph queries showed numerically larger early-phase CMJ gains in the spiral-chain-first group (Pre–Mid mean delta = 3.65 cm vs. 2.80 cm) and numerically larger late-phase gains in the posterior-chain-first group (Post–Follow-up mean delta = 4.49 cm vs. 1.49 cm). These differences were exploratory and were not supported by an independent inferential test of sequence effects across phases.

**Conclusion:**

Myofascial chain training was associated with improvements in CMJ and YBT performance over time, but no training sequence was demonstrated to be statistically superior. The knowledge graph provided a complementary relational representation of changes across intervention phases and individual athlete responses; however, these outputs were descriptive and hypothesis-generating rather than independent inferential evidence. Therefore, the observed numerical differences between sequences should not be interpreted as proof of sequence superiority. Due to the small and specific sample, these findings have limited generalizability, and the observed sequence-specific patterns remain exploratory and hypothesis-generating.

**Trial registration:**

ClinicalTrials.gov NCT07665138, Registered 18/06/2026. Retrospectively registered.

**Supplementary Information:**

The online version contains supplementary material available at https://doi.org/10.1186/s13102-026-02066-0.

## Background

Myofascial structures are biomechanical systems in which muscles, fascia, and connective tissues function as an integrated network, playing a fundamental role in force transmission, movement coordination, and the maintenance of stability throughout the body [1]. They enhance movement efficiency by enabling force transfer between different body segments [2].

Operating within a biotensegrity framework, the muscular and fascial systems form anatomically interconnected meridians (myofascial chains) that transmit forces to distant, non-adjacent structures [3–5]. Rather than being localized to individual muscles, a substantial portion of mechanical force is distributed globally through this intermuscular fascial network [6–8]. In explosive and multidimensional sports like football, athletic actions such as jumping, sprinting, and rapid directional changes heavily rely on this myofascial force transmission and elastic energy storage (the catapult mechanism), and sequential neuromuscular adaptations [2, 9–12]. While specific meridians like the posterior chain primarily drive sagittal-plane explosive power, the lateral and spiral chains govern multi-planar balance, rotational control, and dynamic stabilization [1, 13].

Despite the functional importance of these pathways, targeted myofascial chain training protocols remain remarkably scarce in sports science literature. Existing research is often limited to isolated muscle groups or single-chain protocols, yielding inconsistent performance outcomes; for instance, while anterior chain training has been shown to improve kicking velocity [14], posterior chain protocols have sometimes failed to induce significant jump adaptations [15]. Crucially, the literature has not yet addressed how the sequencing of different myofascial chain interventions affects athletic adaptations over time, nor has it investigated the individual-specific pathways of these adaptations. From this perspective, posterior-chain-first and spiral-chain-first training may plausibly produce different patterns of performance change across the intervention. A posterior-chain-first sequence (PCFG) may initially impose greater mechanical loading on the posterior muscle–tendon system and therefore provide an early mechanical stimulus for subsequent force-production and elastic-function adaptations. Such adaptations are not expected to occur instantaneously, because muscle–tendon remodeling and changes in tissue mechanical properties generally develop progressively in response to repeated loading [16, 17]. In contrast, initiating training with the spiral chain (SCFG) may preferentially challenge multiplanar coordination, dynamic postural control, and intermuscular coordination during the early phase, potentially producing earlier improvements in tasks requiring coordinated whole-body control. Early neural and motor-unit adaptations can occur before substantial structural remodeling is detectable [18, 19]. Conventional analyses estimate overall intervention effects and uncertainty. However, tracking individual athlete profiles over successive time points requires complementary analytical structures. A relational data model may therefore facilitate the organization and exploratory querying of athletes, intervention phases, measurement time points, and performance outcomes within a unified structure. To bridge these gaps, this study investigates the effects of posterior and spiral myofascial chain training on lower-limb explosive power (CMJ) and dynamic balance (YBT) in youth football players (U15–U16) using a crossover design, taking into account the critical periods of biological maturation and physical performance development in adolescent athletes [20, 21]. We hypothesized that targeted myofascial training would improve both performance markers and explored whether the two training sequences were associated with different patterns of change across the intervention. In addition, we examined whether a knowledge graph-based representation could complement the inferential analyses by facilitating descriptive examination of changes across intervention phases and individual athlete trajectories.

Contemporary athlete monitoring involves the repeated collection and interpretation of multidimensional performance data across time. Sport science literature emphasizes that monitoring should account not only for overall group-level changes, but also for individual trends, temporal variation, and the context in which measurements are obtained [22–25]. Athlete monitoring systems similarly aim to integrate multiple performance-related observations within a centralized structure to support the longitudinal interpretation of athlete development [26, 27].

Against this background, a knowledge graph was selected as a suitable complementary data representation for the present study. The dataset contained several explicitly related entities, including athletes, training sequences, intervention phases, measurement time points, performance metrics, and repeated observations. Representing these entities as nodes and their associations as relationships enabled phase-specific and athlete-level observations to be retrieved through a consistent relational structure. Graph- and network-based approaches have previously been applied in sports science to examine interconnected performance variables, and recent sports analytics research has used temporal knowledge graphs to represent football-related entities and events across time [28, 29]. These applications differ from the present intervention study but support the use of graph-based representations when temporal context and explicit relationships among observations are central to the research design.

The knowledge graph was not selected on the assumption that it was statistically superior to conventional methods. Rather, it was used alongside the inferential analyses to organize and descriptively query the temporal and relational structure of the data.

## Methods

**Fig. 1:**
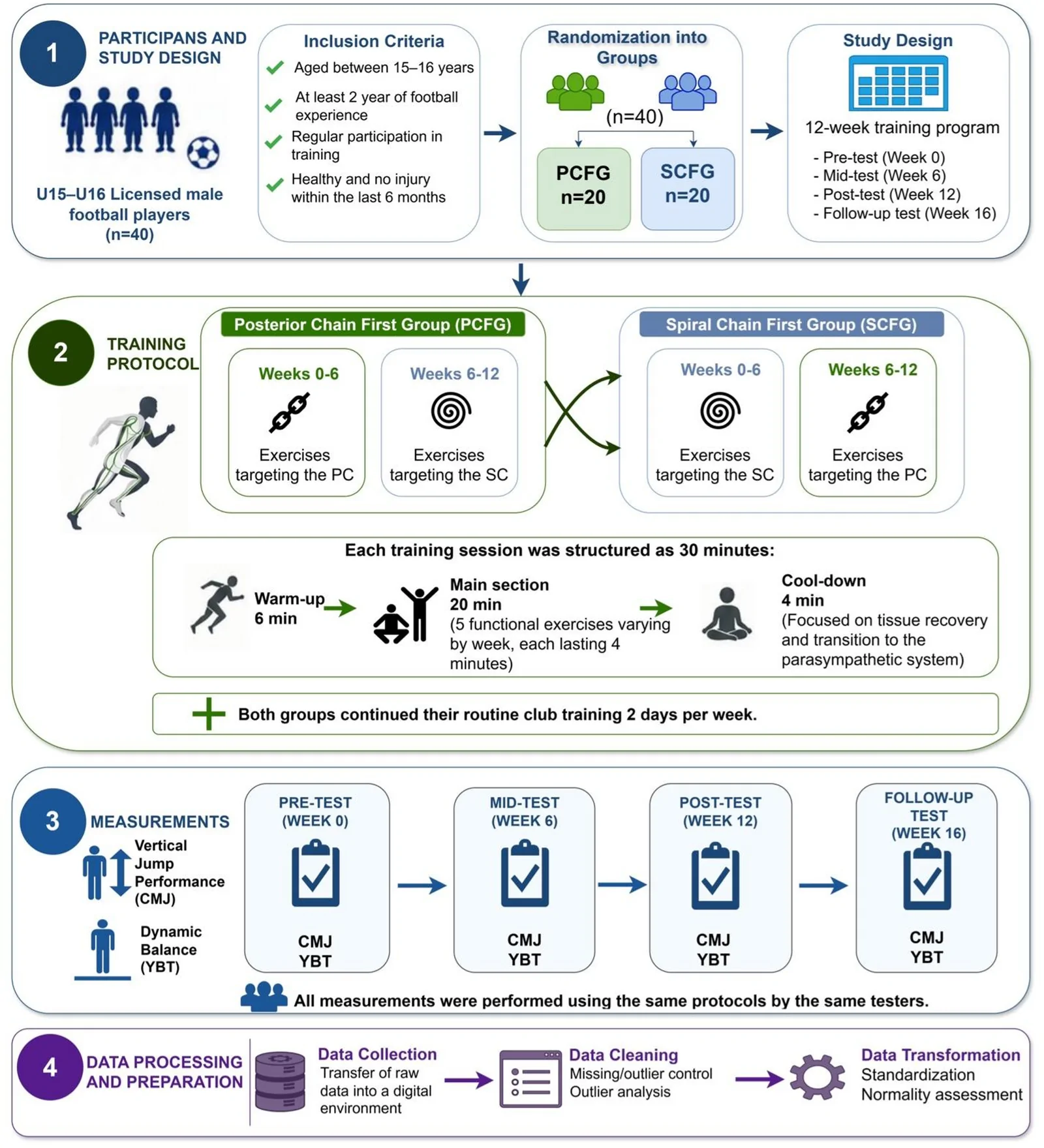
Study design, training protocol, data collection, and preprocessing procedures

In the present study, conventional statistical analyses and a knowledge graph-based analytical framework were integrated and used for complementary purposes to investigate the multidimensional and dynamic nature of performance development. Mixed-design ANOVA and Linear Mixed Model analyses constituted the basis of the inferential evaluation of time, group, and Group × Time effects. The knowledge graph mapped athletes, training sequences, time points, and performance outcomes in a single relational network. It facilitated the retrieval and descriptive examination of phase-specific changes and individual response profiles but was not treated as an independent inferential method. Accordingly, statistical conclusions regarding training-sequence effects were based on the conventional statistical models rather than on the KG-derived descriptive summaries. Parts of the experimental design and data collection procedure were adapted from a previously completed master’s thesis study [30]. Nevertheless, the current study introduces a substantially different analytical framework and presents new findings through knowledge graph-supported analysis. The overall study design, training protocol, data collection procedures, and preprocessing stages are presented in Fig. 1.

### Research design

This study was conducted using a repeated-measures crossover design. This design allows for the comparison of the effects of different training modalities on the same participants, thereby minimizing the influence of inter-individual differences and enabling the acquisition of more reliable results. To ensure objectivity and minimize bias, a single-blind design was implemented; the statistical analysts were kept blinded to the participants’ group allocations and training sequences throughout the entire intervention and evaluation process. However, due to logistical and staffing constraints, the outcome assessor who performed the physical measurements (CMJ and YBT) was not blinded to the participants’ training sequence allocations. Ethical approval for the study was obtained from the Mersin University Sports Sciences Ethics Committee (Decision No: 033, Date: 28.04.2025). Parental consent forms and voluntary informed consent forms were obtained from the parents or legal guardians of all participants.

### Participants

The target population of the study consisted of healthy male football players in the U15 and U16 categories who were licensed and actively participating in training sessions and competitive matches. The sample comprised 40 volunteer football players selected through a convenience sampling method. Since no directly relevant reference study was available for this specific topic, a power analysis was conducted to determine the sample size. The power analysis was performed using G*Power 3.1, with a medium effect size defined as * f=0.25*, a statistical power level of 0.95, and a significance level of $\:\alpha\:=.05$ [31]. The analysis indicated that a minimum of 18 participants per group and at least 36 participants in total would be sufficient. Nevertheless, 40 individuals who voluntarily agreed to participate were included in the study.

After verifying eligibility and obtaining written informed consent, the athletes were randomly allocated (at a 1:1 ratio) to two equal groups (PCFG and SCFG) using the permuted block randomization method via a computer-based random number generator (randomizer.org; available at https://www.randomizer.org). Following the completion of pre-test assessments, the PCFG began a myofascial chain training program focused on the posterior chain muscles, whereas the SCFG initiated a program targeting the spiral chain muscles. Throughout the intervention period, both groups also continued their routine football training sessions. At the end of the first six-week intervention period, mid-test measurements were obtained, after which the groups were crossed over. During the second six-week period, the PCFG performed the spiral chain training protocol, while the SCFG performed the posterior chain training protocol. Upon completion of this phase, post-test measurements were conducted. In order to evaluate the persistence of training effects, a follow-up assessment was performed four weeks after the post-test measurements.

### Data collection instruments and protocols

In this study, vertical jump and dynamic balance measurements were performed to evaluate the participants’ performance characteristics from a multidimensional perspective. Vertical jump performance was considered a fundamental indicator reflecting lower-extremity explosive power capacity, whereas dynamic balance assessments were utilized to evaluate neuromuscular control, proprioceptive feedback, and stabilization capacity. These measurements provide complementary performance indicators capable of revealing the effects of myofascial chain-focused training on both mechanical and neuromotor components. Accordingly, the data collection instruments and application protocols employed in the study are described in detail in this section.

#### Vertical jump performance

Vertical jump performance was measured using a photocell device (SE-JP1). Each participant performed three non-consecutive CMJ within the measurement area of the device while maintaining a stationary position with the hands placed on the hips. To minimize fatigue-related performance fluctuations, a rest interval of 10–15 s was provided between each jump attempt. The mean value of the three trials was used for the analyses [32].

#### Balance performance

Dynamic balance performance was assessed using the Y-Balance Test (YBT). The test was performed barefoot on a dedicated testing platform. Participants stood on one leg behind the starting line and attempted to reach the maximum possible distance in the anterior, posterolateral, and posteromedial directions with the free limb. The testing device was configured with a 135° angle between the anterior and posterior directions and a 90° angle between the two posterior directions. To eliminate potential motor learning effects, and in accordance with the recommendations in the literature [33, 34], six familiarization trials for each lower extremity in all three directions were performed prior to the official assessment. To minimize the effects of fatigue, the measurement sequence was standardized for both lower extremities as anterior, followed by posteromedial and posterolateral directions. Three official trials were performed for each direction. Touching the ground with the free foot, failing to return to the starting position, or removing the hands from the hips were considered errors; such trials were deemed invalid and repeated. The best score obtained in each direction for both lower extremities was included in the analysis. Maximum reach distance was recorded in centimeters (cm) using the measurement scale on the platform, based on the farthest point reached by the distal end of the foot [34]. Lower-extremity length was determined by measuring the distance, in centimeters, between the anterior superior iliac spine and the most distal point of the heel while the participants were in a supine position on a firm surface. All measurements were performed twice by the same researcher, and the mean of the two measurements was accepted as the final value [35]. The abbreviations YBT-R and YBT-L represent dynamic balance performance when the right and left legs, respectively, served as the stance leg. YBT scores were normalized using Eq. (1).

1 $$\:Composite\:Score\:\left(\%\right)=\frac{\left(Anterior+Posteromedial+Posterolateral\right)}{\left(3\times\:Lower\:Extremity\:Length\right)}\times\:100$$

#### Data collection procedure

Following the approval of the ethics committee, pre-test measurements were conducted for all participants included in the study. After the first six-week training period, mid-test assessments were completed, and the groups underwent crossover. At the end of the second six-week intervention period, post-test measurements were performed, followed by follow-up assessments conducted four weeks later. This methodological framework enabled a comprehensive analysis of the temporal changes in training effects. All testing sessions (pre-, mid-, post-test, and follow-up) were conducted at the same time of day and on the same indoor testing surface for each participant, in order to minimize circadian and surface-related variability. Pre-testing fatigue and nutritional status were not systematically standardized or monitored, which we acknowledge as a limitation of the testing protocol (see Limitations).

### Training program

Each training session (Additional files 1 and 2) was structured to last 30 min and consisted of a warm-up phase (6 min), a main phase (20 min; including five functional exercises lasting 4 min each and varying according to the training week), and a cool-down phase (4 min; aimed at tissue recovery and transition to parasympathetic activation). In addition to the myofascial chain training sessions, both groups continued their routine club training twice per week (which consisted of standardized tactical and technical drills with matched volumes and intensities for all players, avoiding additional strength or explosive loading). All components of the training program were selected and adapted from the book by [36]. Myofascial chain sessions were delivered twice weekly (12 sessions per 6-week phase). Within each 6-week block, exercises progressed across three 2-week sub-blocks (weeks 1–2, 3–4, 5–6): the initial sub-block emphasized mobility and movement-pattern familiarization (“feel-it” exercises), the second introduced sustained stretch-based loading of the target chain, and the third incorporated dynamic, elastic-recoil-oriented exercises (e.g., diagonal lunges, boomerang-type movements). Progression was applied uniformly by training week rather than individualized to athlete-specific readiness; formal RPE or %MVC-based intensity monitoring was not implemented, meaning that individual variations in the experienced physiological stimulus could not be controlled. We fully acknowledge this lack of individualized load control as a methodological limitation (see Limitations). To ensure consistency, all myofascial training sessions were delivered by the same qualified specialist, and protocol fidelity was systematically verified at each session using a standardized photographic execution checklist. Attendance was monitored by the supervising coach at every session; athletes were permitted a maximum of two excused absences per 12-week phase, and no participant exceeded this threshold. The Posterior Chain protocol emphasized plantar fascia release, hip-hinge, and elastic-recoil exercises (e.g., pogo jumps, hip thrusts, hamstring bridges) targeting the superficial back line, whereas the Spiral Chain protocol emphasized rotational mobility and cross-body loading exercises (e.g., standing and supine corkscrews, diagonal lunges, rotational split squats) targeting the spiral line. Within each 6-week phase, exercises progressed across three 2-week sub-blocks, moving from mobility- and pattern-familiarization exercises (weeks 1–2), to sustained stretch-based loading (weeks 3–4), to dynamic, elastic-recoil-oriented exercises (weeks 5–6), with exercise selection and loading criteria adapted from Albini [36]. Full session-by-session details, including exercise names, durations, repetitions, and execution cues, are provided in Additional files 1 and 2.

### Statistical analysis

**Fig. 2:**
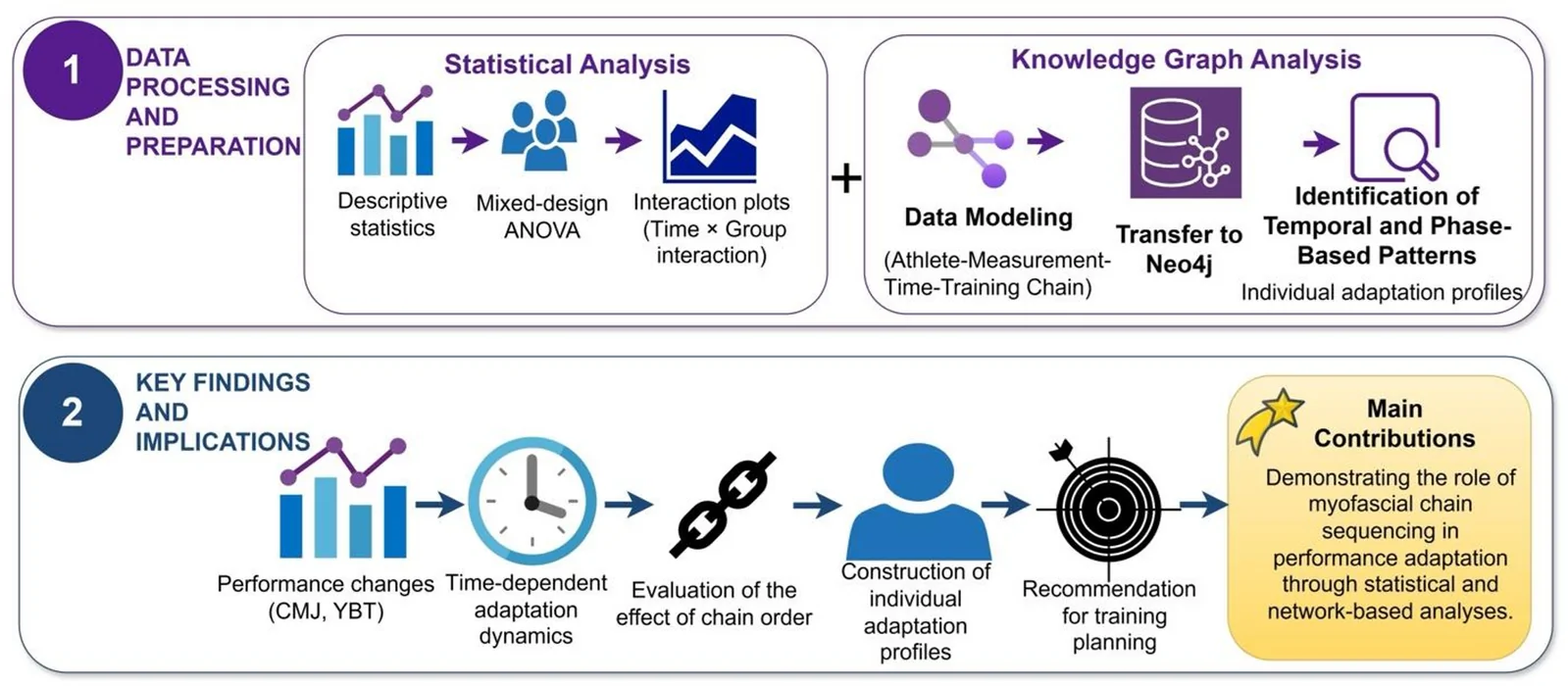
Data processing and analysis procedures

This study was conducted and reported in accordance with the Consolidated Standards of Reporting Trials (CONSORT) guidelines. The data processing and analysis procedures employed in the study are presented in Fig. 2.

The normality of the data distribution was assessed using the Shapiro–Wilk test. The results indicated that the assumption of normality was satisfied at all measurement time points (pre-test, mid-test, post-test, and follow-up test) for both groups (* p>.05*). This finding was further supported by the examination of skewness and kurtosis coefficients.

For the analysis of the CMJ, YBT-R, and YBT-L variables, a 2 × 4 Mixed-Design ANOVA was conducted to compare performance changes between the two groups across four different measurement time points [37]. The assumption of sphericity was evaluated using Mauchly’s test, and the Greenhouse–Geisser correction was applied when violations of sphericity were detected. In cases where the main effect of time was found to be statistically significant, Bonferroni-adjusted pairwise comparisons were performed to determine between which measurement time points the differences occurred. Effect sizes were reported using partial eta squared ($\:{\eta\:}_{p}^{2}$). Statistical significance was accepted at * p<.05*for all analyses. To assess potential carryover effects, a mixed-design ANOVA was performed for each outcome parameter, with phase gain score as the within-subject factor and group sequence (PCFG, SCFG) as the between-subject factor; a non-significant Phase × Group interaction (*p* > .05) was used as the criterion for the absence of carryover. To visualize the developmental patterns of the groups over time, an interaction plot was generated within the framework of the Mixed-Design ANOVA. In this plot, the vertical axis represented the estimated marginal means, whereas the horizontal axis represented the four measurement time points. The two groups were represented using different colors and symbols, allowing the parallelism of change patterns and the presence of interaction effects to be visually evaluated. In addition to the 2 × 4 mixed-design ANOVA, we performed a Linear Mixed Model (LMM) analysis with restricted maximum likelihood estimation to account for the repeated-measures structure.

### Construction of the knowledge graph-based data model

Knowledge graph approaches are advanced data modeling methodologies that represent relationships among entities through interconnected nodes and edges, thereby providing a powerful analytical framework for the representation of multidimensional and relational data structures. The application of graph-based approaches in sports science has only recently begun to gain broader scholarly attention and methodological traction [38–40]. The selection of a knowledge graph was guided by the relational and longitudinal structure of the study data and by the intended exploratory queries. Each athlete was associated with repeated observations across four measurement time points, two intervention phases, a crossover training sequence, and three performance outcomes. The graph representation retained these relationships explicitly and allowed observations to be traced from an athlete to the relevant training phase, chain type, time point, and performance metric. This structure facilitated consistent retrieval of consecutive phase changes and athlete-level longitudinal profiles without altering the original relationships among the study entities.

The knowledge graph should not be considered the only possible representation of these data. Conventional relational or long-format tabular structures could also be used to store and analyze the same observations. No formal comparison of database architectures or query performance was conducted. Therefore, the KG was regarded as a suitable complementary representation for the specific relational and exploratory objectives of the study, rather than as a demonstrably superior data-management or analytical method [41]. In the present study, the dataset was modeled as a knowledge graph to facilitate the relational representation and descriptive exploration of temporal performance changes, training-sequence-related patterns, and inter-individual variability in youth football players. The knowledge graph model was constructed using the Neo4j graph database (Neo4j, Inc, San Mateo, CA, USA) platform in order to establish a relational and temporal organization of the dataset. Within this framework, the graph-based modeling approach enabled the integration of athletes, applied myofascial chain interventions, measurement time points, and performance-related parameters into a unified analytical structure. Consequently, multidimensional relationships among training sequence, time-course of improvements, and athlete-specific developmental profiles could be descriptively examined within a relational structure alongside the conventional tabular and statistical analyses.

**Fig. 3:**
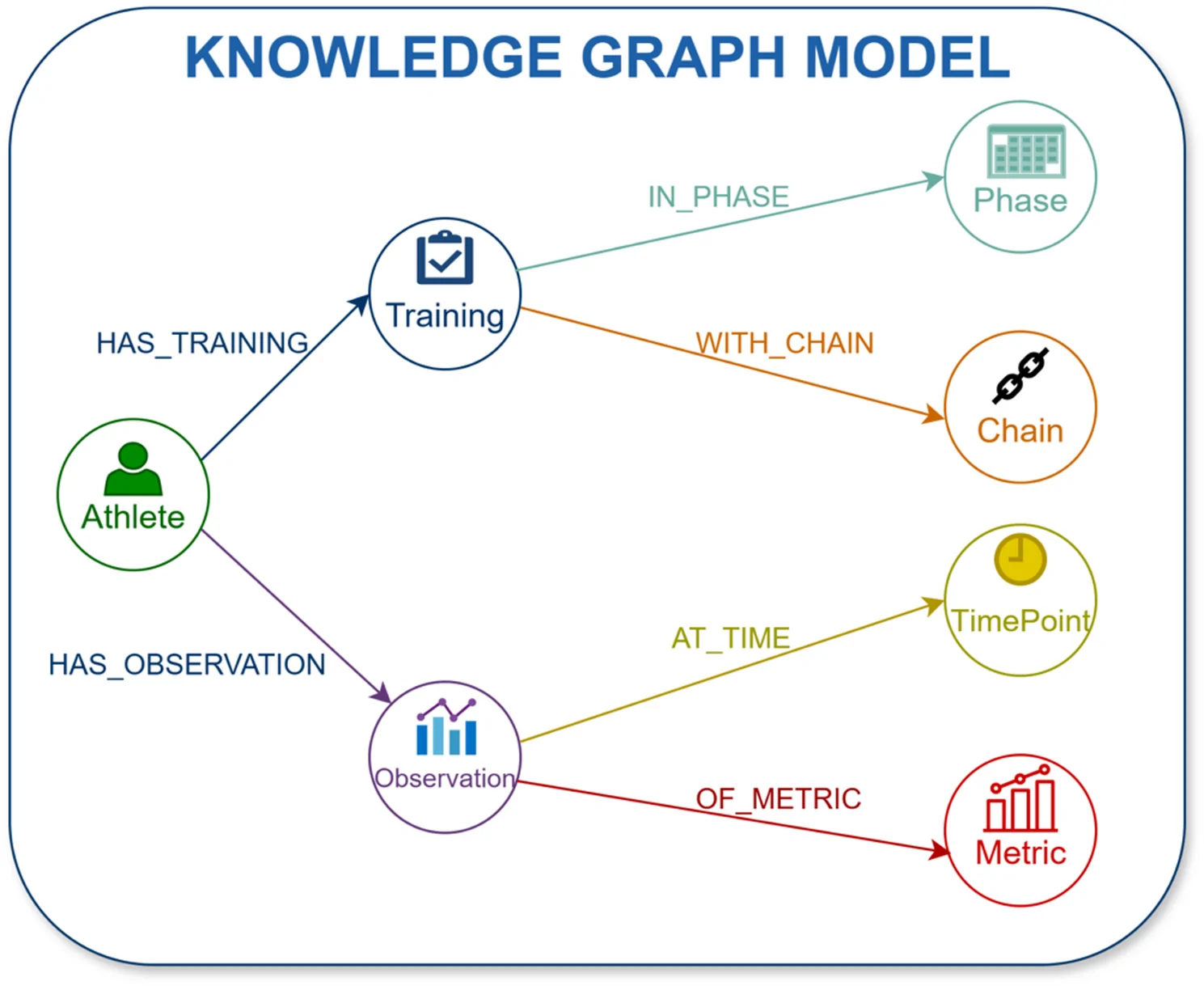
Conceptual schema of the Neo4j knowledge graph used to represent the study data. Note: Circles represent node types, arrowed lines indicate the direction of relationships, and uppercase labels denote relationship types. Each Athlete node is connected to one or more Training nodes through HAS_TRAINING and to repeated Observation nodes through HAS_OBSERVATION. Each training record is linked to its corresponding intervention Phase and myofascial Chain through IN_PHASE and WITH_CHAIN, respectively. Each observation is linked to the relevant measurement TimePoint through AT_TIME and to the assessed performance Metric through OF_METRIC. Phase represents the intervention phase; Chain represents posterior- or spiral-chain training; TimePoint represents Pre-test, Mid-test, Post-test, or Follow-up; and Metric represents CMJ, YBT-R, or YBT-L. Colors are used only to distinguish node and relationship types

The knowledge graph was constructed using four primary data sources: observations, phases, athletes, and assignments. Athletes, phases, and chain types were initially defined as independent nodes, after which Training and Observation nodes were integrated to represent intervention contexts and measurement records within a unified relational structure. This framework enabled temporal changes, training-sequence-related patterns, and performance parameters to be retrieved and descriptively examined within an explicitly connected data structure. A representative example of the proposed graph structure is presented in Fig. 3. Within this framework, Athlete nodes formed the core of the model and were directly connected to measurement and training contexts through systematically defined relationships. Consequently, performance outcomes could be interpreted together with their temporal and contextual dimensions, facilitating the descriptive examination of phase-specific patterns and athlete-level developmental trajectories.

#### Knowledge graph implementation and computational reproducibility

The knowledge graph was implemented using Neo4j AuraDB in Windows 10. The implementation was based on a predefined graph schema comprising Athlete, Training, Chain, Phase, Observation, TimePoint, and Metric nodes. The principal relationship types were HAS_TRAINING, WITH_CHAIN, IN_PHASE, HAS_OBSERVATION, AT_TIME, and OF_METRIC. Uniqueness constraints and indexes were defined before data import to ensure consistent node identification and prevent duplicate records.

For each athlete and outcome, the phase-specific change score (Δ) was calculated by subtracting the value at the earlier measurement time point from the value at the subsequent measurement time point. Accordingly, separate change scores were calculated for the Pre-test–Mid-test, Mid-test–Post-test, and Post-test–Follow-up transitions. Mean Delta represents the arithmetic mean of the individual athlete change scores within a training-sequence group, SD Delta represents the standard deviation of these individual change scores, and Median Delta represents their median. SD Delta does not represent the difference between the standard deviations recorded at the two measurement time points. Total Gain was calculated for each athlete as the Follow-up value minus the Pre-test value.

The computational workflow consisted of data preprocessing, graph construction, relational validation, phase-based analysis, athlete-level analysis, and result export. During preprocessing, the longitudinal performance records were transformed into a structured format compatible with the Neo4j import procedure. The input structure included pseudonymous athlete codes, training-sequence assignments, measurement time points, performance metrics, and observed values. The populated database itself was not used to store names, contact details, or other direct identifiers. Athlete-level queries subsequently ranked participants according to their total CMJ, YBT-R, and YBT-L gains.

All schema definitions, constraints, indexes, data-import templates, analytical Cypher queries, preprocessing scripts are available as the supplementary material. The original participant-level dataset and populated Neo4j database are not publicly available because the participants were minors and the longitudinal records contain sensitive individual-level information. The combination of demographic, intervention, and repeated performance variables may enable participant re-identification even after removal of direct identifiers. Therefore, the shared reproducibility package supports transparent examination and reimplementation of the computational workflow but does not permit independent regeneration of the reported numerical results from the confidential original dataset.

In the present study, the knowledge graph was used as a complementary relational and exploratory framework rather than as an independent inferential model. Statistical conclusions regarding time, group, and training-sequence effects were based on the mixed-design ANOVA and Linear Mixed Model analyses. The KG queries were used to retrieve linked observations, calculate descriptive changes between consecutive measurement time points, and examine athlete-level longitudinal profiles. Because the KG-derived summaries did not independently estimate statistical uncertainty, p-values, confidence intervals, or effect sizes, they were not interpreted as evidence of training-sequence superiority. Furthermore, the KG was not formally benchmarked against conventional statistical or tabular workflows in terms of predictive accuracy, model fit, statistical power, or computational efficiency.

## Results

The mean height of the participants was measured as $\:166.75\pm\:10.30\:$cm in the PCFG group and $\:168.95\pm\:12.03\:$cm in the SCFG group, while the mean body weight was $\:51.84\pm\:7.29\:$kg for the PCFG group and $\:56.89\pm\:11.49\:$kg for the SCFG group. Independent samples t-test results conducted to compare pre-intervention characteristics, including height, body weight, and sports license duration, revealed no statistically significant differences between the groups for any of the examined variables (* p>.05*). These findings indicate that the PCFG and SCFG groups exhibited a comparable and homogeneous structure in terms of baseline physical characteristics and sports background, thereby providing an appropriate basis for intergroup comparisons.

Descriptive values are presented in Table 1. CMJ, YBT-R, and YBT-L scores increased from Pre-test to Follow-up in both groups, with broadly similar overall trajectories.

**Table 1:** Descriptive statistics for CMJ and YBT performance across groups and measurement time points

Tests	Groups	CMJ (cm)^a^	YBT-*R* (%) ^a^	YBT-L (%) ^a^
Pre-test	PCFG	36.12 ± 7.68	78.4 ± 9.34	78.69 ± 7.95
	SCFG	35.27 ± 7.39	78.38 ± 9.00	79.59 ± 7.86
Mid-test	PCFG	38.92 ± 8.24	79.38 ± 9.18	79.76 ± 7.74
	SCFG	38.92 ± 10.88	80.12 ± 8.77	80.53 ± 8.02
Post-test	PCFG	41.76 ± 8.52	84.58 ± 11.75	83.42 ± 9.88
	SCFG	40.81 ± 10.19	82.73 ± 8.08	82.83 ± 10.07
Follow-up test	PCFG	44.25 ± 8.64	86.43 ± 10.93	84.5 ± 9.26
	SCFG	42.29 ± 9.86	83.88 ± 8.21	84.03 ± 9.84

Pre-test = assessment conducted before the intervention; Mid-test = assessment conducted after the first 6-week intervention phase; Post-test = assessment conducted after the second 6-week intervention phase; Follow-up = assessment conducted four weeks after the Post-test. CMJ values are expressed in centimeters; YBT scores are expressed as normalized composite percentages (%)

*PCFG* posterior-chain-first group, *SCFG* spiral-chain-first group, *CMJ* countermovement jump, *YBT-R* Y-BalanceTest score with the right leg as the stance leg, *YBT-L* Y-Balance Test score with the left leg as the stance leg, *SD* standard deviation

^a^ Values are presented as mean ± SD

Mixed-design ANOVA showed a significant main effect of time for CMJ, YBT-R, and YBT-L, but no significant Time × Group interaction for any outcome (Table 2). Thus, performance improved over time without inferential evidence that either training sequence produced a superior overall trajectory.

**Table 2:** Mixed-Design ANOVA Results for CMJ, YBT-R, YBT-L Scores across Measurement Times and Groups (Greenhouse-Geisser)

Source		Type III Sum of Squares	df	Mean Square	F	*p*	ηp²
CMJ	**Time**	1273.278	1.515	840.284	69.145	< 0.001	0.645
	*Time x Group*	19.195	1.515	12.667	1.042	0.342	0.027
	*Error(Time)*	699.757	57.581	12.153			
YBT-R	**Time**	1221.320	1.033	1181.888	17.123	< 0.001	0.311
	*Time x Group*	70.905	1.033	68.616	0.994	0.328	0.025
	*Error(Time)*	2710.326	39.268	69.022			
*YBT-L*	***Time***	703.576	1.052	668.859	11.448	< 0.001	0.232
	*Time x Group*	18.911	1.052	17.978	0.308	0.594	0.008
	*Error(Time)*	2335.496	39.972	58.428			

Greenhouse–Geisser-corrected results are reported where the assumption of sphericity was violated. The within-subject factor Time comprised four measurement points: Pre-test, Mid-test, Post-test, and Follow-up. The between-subject factor Group comprised PCFG and SCFG

*PCFG* posterior-chain-first group, *SCFG* spiral-chain-first group, *CMJ* countermovement jump, *YBT-R* Y-Balance Test score with the right leg as the stance leg, *YBT-L* Y-Balance Test score with the left leg as the stance leg, df degrees of freedom, *F*  F-statistic, *p* probability value, ηp² partial eta squared

Statistical significance was set at *p* < .05

Bonferroni-adjusted comparisons showed significant differences between all CMJ measurement points and between all YBT-R measurement points. For YBT-L, all comparisons were significant except the Mid-test–Post-test comparison. The largest changes for all three outcomes occurred between Pre-test and Follow-up; the estimated differences and 95% confidence intervals are reported in Table 3.

**Table 3:** Bonferroni-adjusted pairwise comparisons across measurement times for CMJ, YBT-R, and YBT-L

Measurements	(I) Time	(J) Time	Mean Difference (I-J)	Std. Error	p^b^	95% Confidence Interval for Difference^b^	
						Lower Bound	Upper Bound
CMJ	1	2	-3.225^*^	0.750	< 0.001	-5.312	-1.138
		3	-5.585^*^	0.722	< 0.001	-7.594	-3.575
		4	-7.572^*^	0.679	< 0.001	-9.463	-5.680
	2	1	3.225^*^	0.750	< 0.001	1.138	5.312
		3	-2.360^*^	0.285	< 0.001	-3.154	-1.566
		4	-4.347^*^	0.405	< 0.001	-5.475	-3.219
	3	1	5.585^*^	0.722	< 0.001	3.575	7.594
		2	2.360^*^	0.285	< 0.001	1.566	3.154
		4	-1.987^*^	0.226	< 0.001	-2.615	-1.359
	4	1	7.572^*^	0.679	< 0.001	5.680	9.463
		2	4.347^*^	0.405	< 0.001	3.219	5.475
		3	1.987^*^	0.226	< 0.001	1.359	2.615
YBT-R	1	2	-1.359^*^	0.160	< 0.001	-1.805	− 0.914
		3	-5.266^*^	1.366	0.003	-9.068	-1.464
		4	-6.767^*^	1.324	< 0.001	-10.453	-3.082
	2	1	1.359^*^	0.160	< 0.001	0.914	1.805
		3	-3.907^*^	1.337	0.035	-7.627	− 0.186
		4	-5.408^*^	1.291	< 0.001	-9.001	-1.815
	3	1	5.266^*^	1.366	0.003	1.464	9.068
		2	3.907^*^	1.337	0.035	0.186	7.627
		4	-1.501^*^	0.189	< 0.001	-2.027	− 0.976
	4	1	6.767^*^	1.324	< 0.001	3.082	10.453
		2	5.408^*^	1.291	< 0.001	1.815	9.001
		3	1.501^*^	0.189	< 0.001	0.976	2.027
YBT-L	1	2	-1.004^*^	0.239	< 0.001	-1.670	− 0.339
		3	-3.988^*^	1.195	0.011	-7.313	− 0.663
		4	-5.125^*^	1.160	< 0.001	-8.356	-1.895
	2	1	1.004^*^	0.239	< 0.001	0.339	1.670
		3	-2.983	1.300	0.164	-6.601	0.634
		4	-4.121^*^	1.262	0.014	-7.635	− 0.607
	3	1	3.988^*^	1.195	0.011	0.663	7.313
		2	2.983	1.300	0.164	− 0.634	6.601
		4	-1.138^*^	0.180	0.001	-1.639	− 0.637
	4	1	5.125^*^	1.160	< 0.001	1.895	8.356
		2	4.121^*^	1.262	0.014	0.607	7.635
		3	1.138^*^	0.180	< 0.001	0.637	1.639

*Abbreviation* Comparisons are based on estimated marginal means. Time codes are defined as follows: 1 = Pre-test, 2 = Mid-test, 3 = Post-test, and 4 = Follow-up. The mean difference was calculated as the estimated marginal mean at Time I minus the estimated marginal mean at Time J. *CMJ* countermovement jump, *YBT-R* Y-Balance Test score with the right leg as the stance leg, *YBT-L* Y-Balance Test score with the left leg as the stance leg, *SE* standard error, *CI* confidence interval, *p* Bonferroni-adjusted probability value

^*^The mean difference is significant at the 0.05 level

^b^Adjustment for multiple comparisons: Bonferroni

**Fig. 4:**
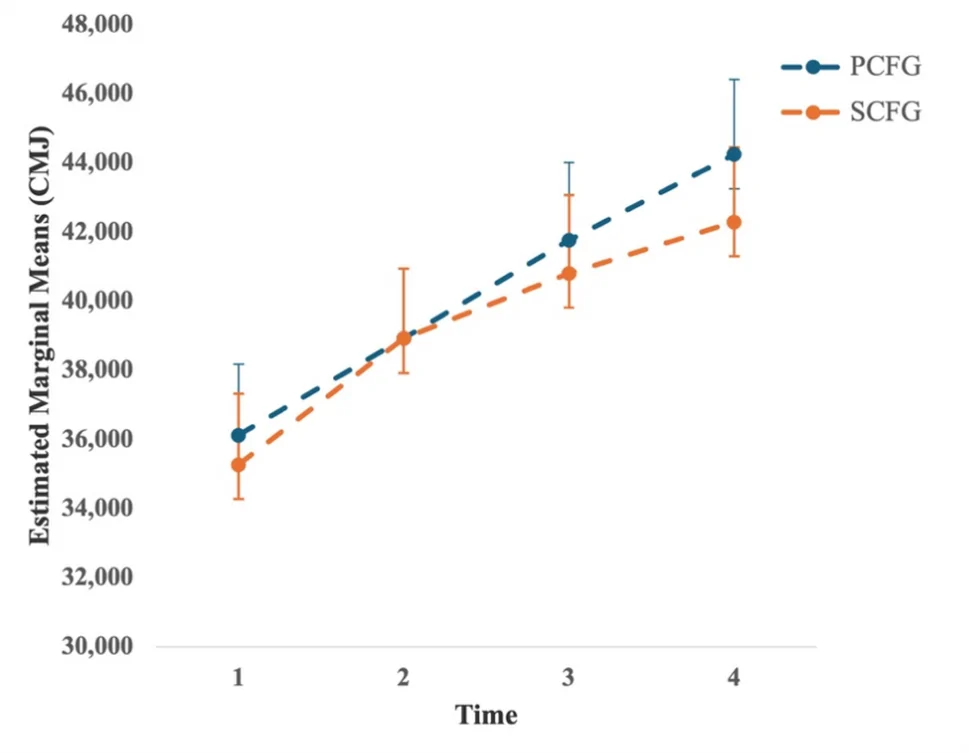
Change Profile of Estimated Marginal Means for CMJ Scores Across Measurement Times by PCFG and SCFG Groups. Note: Vertical error bars indicate ± 1 SD

**Fig. 5:**
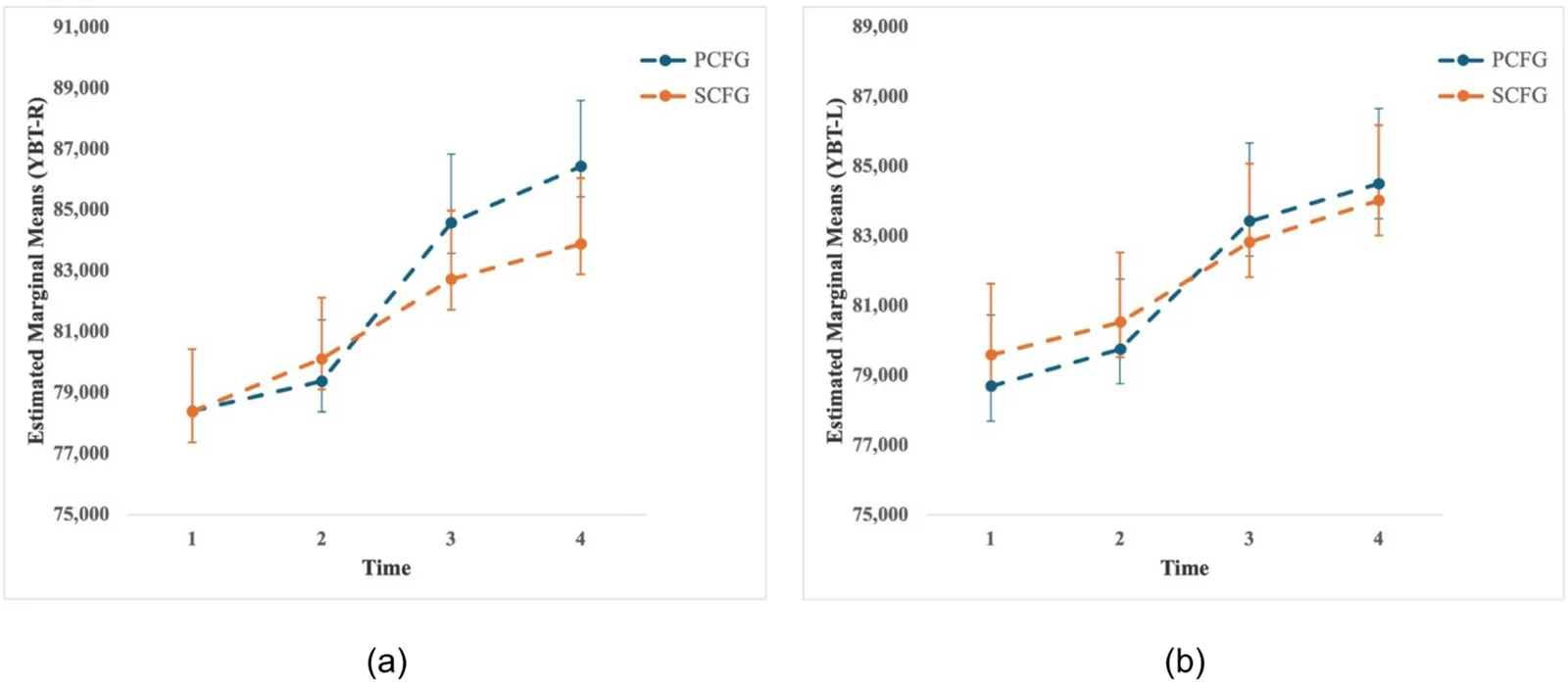
(**a**) Change Profile of Estimated Marginal Means for YBT-R Scores Across Measurement Times by PCFG and SCFG Groups (**b**) Change Profile of Estimated Marginal Means for YBT-L Scores Across Measurement Times by PCFG and SCFG Groups (1 = pre-test, 2 = mid-test, 3 = post-test, 4 = follow-up test) Note: Vertical error bars indicate ± 1 SD

The profile plots in Figs. 4 and 5a and b illustrate the broadly parallel group trajectories underlying the non-significant Time × Group interactions.

No significant Phase × Group interaction was detected for CMJ, YBT-R, or YBT-L, indicating no statistically detectable carryover effect (Table 4). To further account for the repeated-measures structure, a Linear Mixed Model analysis was performed. The LMM confirmed a significant main effect of time for CMJ, YBT-R, and YBT-L (*p* < .001 for all outcomes). A significant Group × Time interaction was observed only for YBT-R [F(3,38) = 4.900, *p* = .006], indicating an outcome-specific difference in the temporal trajectory of right-side dynamic balance. The discrepancy between the two inferential models regarding YBT-R—where the LMM identified a significant interaction while the mixed-design ANOVA did not—is primarily attributable to their differing treatment of repeated-measures covariance and inter-individual variability. While mixed-design ANOVA assumes compound symmetry and uses a pooled error term (Greenhouse-Geisser corrected), LMM models subject-specific random intercepts. By accounting for the high baseline heterogeneity and athlete-specific response profiles inherent in dynamic balance, the difference may reflect the models’ different treatment of repeated-measures covariance and inter-individual variability. This result does not establish the overall superiority of either training sequence. The Group × Time interactions were not statistically significant for CMJ [F(3,38) = 2.233, *p* = .100] or YBT-L [F(3,40.902) = 0.238, *p* = .870]. Accordingly, the phase-specific KG patterns, particularly for CMJ and YBT-L, were interpreted descriptively rather than as inferential evidence of sequence effects (Tables 6 and 7).

**Table 4:** Assessment of potential carryover effects based on the Phase × Training-Sequence Group interaction

Parameter	F	df	*p*	ηp²	Carryover?
CMJ	1.061	1	0.309	0.027	No
YBT-R	1.589	1	0.215	0.040	No
YBT-L	0.186	1	0.669	0.005	No

Phase 1 gain was calculated as Mid-test minus Pre-test, and Phase 2 gain was calculated as Post-test minus Mid-test. The within-subject factor was intervention phase, and the between-subject factor was training-sequence group. *PCFG* posterior-chain-first group, *SCFG* spiral-chain-first group, *CMJ* countermovement jump, *YBT-R* Y-Balance Test score with the right leg as the stance leg, *YBT-L* Y-Balance Test score with the left leg as the stance leg, *F* F-statistic, *df* degrees of freedom, *p* probability value, *ηp²* partial eta squared. A non-significant Phase × Group interaction (*p* > .05) was interpreted as no statistically detectable carryover effect

### Exploratory descriptive findings obtained through the knowledge graph

Following the inferential analyses, the knowledge graph was queried to summarize phase-specific changes and athlete-level response patterns. The results in this section are descriptive and hypothesis-generating; all inferential conclusions are based on the mixed-design ANOVA and LMM analyses.

#### Vertical jump analyses

The KG summaries showed a numerically larger Pre-test–Mid-test mean change in SCFG, whereas PCFG showed numerically larger Mid-test–Post-test, Post-test–Follow-up, and total changes (Table 5). The dispersion of the change scores also indicated marked inter-individual variability. Because the CMJ Group × Time interaction was not significant, these differences are interpreted descriptively rather than as evidence of sequence superiority. Therefore, individual athlete developments were further examined within the CMJ analysis, and the results obtained for the five athletes with the highest and lowest total gains are presented in Supplementary Table S1.

**Table 5:** KG-derived descriptive phase changes and total gains in CMJ by training sequence

	Pre-test–Mid-test Transition	Mid-test–Post-test Transition	Post-test–Follow-up Test Transition	Pre-test–Follow-up Test Transition
	Mean Δ ± SDΔ	Mean Δ ± SDΔ	Mean Δ ± SDΔ	Mean Total Gain ± SD
PCFG	2.80 ± 2.66	**2.84** ± 1.63	**4.49** ± 1.26	**8.12** ± 3.97
SCFG	**3.65** ± **5.97**	1.89 ± **1.88**	1.49 ± **1.51**	7.02 ± **4.40**

For each phase transition, the individual change score (Δ) was calculated as the later CMJ value minus the earlier CMJ value. Mean Δ represents the arithmetic mean of the individual change scores within the relevant training-sequence group, and SDΔ represents the standard deviation of those individual change scores. Total Gain was calculated for each athlete as Follow-up CMJ minus Pre-test CMJ; the table presents the group mean and standard deviation of these individual total-gain values. SDΔ does not represent the difference between the standard deviations at the two measurement time points. PCFG posterior-chain-first group, corresponding to the PC→SC sequence; *SCFG* spiral-chain-first group, corresponding to the SC→PC sequence, *PC* posterior chain, *SC* spiral chain, *CMJ* countermovement jump. All values are expressed in centimeters. Positive values indicate improvement. These values are descriptive and do not represent inferential comparisons between the training sequences

Examination of individual performance improvements revealed substantial variability among athletes. The maximum observed gain was approximately 19.81, whereas the minimum gain was approximately 1. Although high levels of improvement were observed in athletes from both chain-sequencing conditions, the broad distribution of total gains indicates that the observed variability in performance changes was not clearly attributable to chain sequence alone. Among the ten athletes demonstrating the highest gains, 40% initially performed the spiral chain (SC) protocol, whereas 60% began with the posterior chain (PC) protocol. In contrast, analysis of the ten athletes with the lowest gains revealed that 70% had started with the SC protocol, while only 30% had begun with the PC protocol (Supplementary Table S1).

Positive mean changes were observed across the YBT-R phases in both sequences (Table 6). For YBT-R, SCFG showed a numerically larger Pre-test–Mid-test mean change, whereas PCFG showed larger mean changes during the subsequent transitions (Table 6). Although the LMM identified an overall Group × Time interaction, no phase-specific inferential contrasts were performed. The KG-derived phase differences are therefore presented descriptively and do not identify a superior sequence (Table 7).

**Table 6:** KG-derived descriptive phase changes in YBT-R by training sequence

Phase	Chain Sequence	Mean Δ	SD Δ	Median Δ
Pre-test–Mid-test	PCFG	0.98	0.69	1.01
	SCFG	1.74	1.21	1.65
Mid-test–Post-test	PCFG	5.20	9.86	3.22
	SCFG	2.61	6.22	1.80
Post-test–Follow-up Test	PCFG	1.86	1.48	1.57
	SCFG	1.15	0.72	1.14

For each athlete and phase transition, Δ was calculated as the normalized YBT-R score at the later measurement time point minus the score at the earlier measurement time point. Mean Delta represents the arithmetic mean of the individual change scores within the relevant training-sequence group; SD Delta represents the standard deviation of those individual change scores; and Median Delta represents the median of those individual change scores. SD Delta does not represent the difference between the standard deviations at the two measurement time points. *YBT-R* Y-Balance Test score with the right leg as the stance leg, *PCFG* posterior-chain training followed by spiral-chain training, *SCFG* spiral-chain training followed by posterior-chain training, *PC* posterior chain, *SC* spiral chain. Delta values are expressed in percentage points. Positive values indicate improvement. These values are descriptive and do not represent inferential comparisons between training sequences

**Table 7:** KG-derived descriptive phase changes in YBT-L by training sequence

Phase	Chain Sequence	Mean Δ	SD Δ	Median Δ
Pre-test–Mid-test	PCFG	1.07	0.61	1.04
	SCFG	0.94	2.00	1.40
Mid-test–Post-test	PCFG	3.67	7.43	1.64
	SCFG	2.30	8.55	0.46
Post-test–Follow-up Test	PCFG	1.08	1.04	0.85
	SCFG	1.20	1.18	1.02

For each athlete and phase transition, Δ was calculated as the normalized YBT-L score at the later measurement time point minus the score at the earlier measurement time point. Mean Delta represents the arithmetic mean of the individual change scores within the relevant training-sequence group; SD Delta represents the standard deviation of those individual change scores; and Median Delta represents the median of those individual change scores. SD Delta does not represent the difference between the standard deviations at the two measurement time points. *YBT-L*  Y-Balance Test score with the left leg as the stance leg, *PCFG* posterior-chain training followed by spiral-chain training, *SCFG* spiral-chain training followed by posterior-chain training, *PC* posterior chain, *SC* spiral chain. Delta values are expressed in percentage points. Positive values indicate improvement. These values are descriptive and do not represent inferential comparisons between training sequences

Individual YBT gains varied widely, including negative changes in both sequences. High and low responders were observed in each group, supporting heterogeneous athlete-specific responses rather than a uniform sequence effect (Supplementary Table S2 and Table S3).

## Discussion

This study investigated how different myofascial chain training sequences affected CMJ and YBT performance in youth football players. It also examined how performance changed across successive measurement periods. Both groups improved significantly over time, regardless of the training sequence. For CMJ, all measurement points differed significantly, and the largest change occurred between the initial and final assessments. Conventional statistical analyses did not identify significant overall differences between the groups. The Neo4j-based knowledge graph provided an additional descriptive view of the Pre-test–Mid-test, Mid-test–Post-test, and Post-test–Follow-up transitions. It also allowed sequence-related patterns to be examined alongside the inferential analyses. Performance changes should therefore be considered not only in terms of total improvement but also in relation to when those changes occurred.

The more rapid CMJ improvement observed in the SCFG group during the Pre-test–Mid-test phase represented a numerically greater phase-specific change; however, the Group × Time interaction for CMJ was not statistically significant. Therefore, this difference should be interpreted as a descriptive temporal pattern rather than evidence of a sequence-specific early effect. Early improvements in jumping performance may involve task-specific learning or neuromuscular processes [18, 19, 42]; however, these mechanisms were not directly assessed in the present study and therefore cannot be established from the available data. By contrast, the exploratory knowledge graph summaries showed numerically larger CMJ gains for the posterior-chain-first sequence from Mid-test to Post-test and during the Follow-up period. However, neither the conventional ANOVA nor the LMM demonstrated a significant Time × Group interaction for CMJ. Therefore, these sequence-related differences should be regarded as descriptive and hypothesis-generating rather than as evidence of differential physiological adaptation.

The relatively high CMJ standard deviations at the end of the intervention indicate marked variability among athletes. The Neo4j analyses further showed that the observed distribution reflected distinct athlete-level response profiles. Large gains occurred in both sequencing conditions, suggesting that individual responsiveness was more influential than group averages alone. Such variability may reflect differences in intrinsic biomechanical and morphological characteristics [43]. Connective-tissue responses to mechanical loading, particularly tendon responses, vary considerably among individuals. Baseline mechanical properties, anthropometric and body-composition characteristics, and previous loading or training exposure may contribute to this variability [17, 44–46]. Among the ten athletes with the greatest CMJ gains, 60% began with the PC sequence. The Neo4j summaries also showed larger numerical gains among some posterior-chain-first athletes during the Post-test–Follow-up phase and across the overall Pre-test–Follow-up period; however, these observations remain descriptive and should not be interpreted as evidence of a sequence-specific advantage.

CMJ performance depends on coordinated stretch–shortening behavior within the lower-limb muscle–tendon units. During the countermovement, some muscle fascicles may behave quasi-isometrically while aponeurotic and tendinous tissues store elastic strain energy. During push-off, the fascicles shorten actively and the elastic tissues recoil, contributing to muscle–tendon unit power and jump performance [47–50]. Efficient tendon energy return is associated with low hysteresis. Changes in tendon stiffness and Young’s modulus generally require several weeks of repeated loading at a sufficiently high strain and may develop more slowly than early neural responses [17, 18, 51]. Beginning the intervention with PC training may have provided an earlier mechanical stimulus to the muscle–tendon system, although this interpretation remains speculative because these tissue-level changes were not directly measured [52]. Conversely, 70% of the ten athletes with the smallest CMJ gains began with the SC sequence. This pattern suggests that neural responses without sufficient structural adaptation may limit longer-term improvement [53]. When PC training was introduced later, the remaining intervention period may not have provided enough cumulative mechanical stimulus to induce hypertrophy or material changes in tendons and aponeuroses [16]. High-performing athletes were observed in both training sequence groups, indicating that CMJ responses varied across individuals and were not consistently differentiated by training sequence. This variability may also be interpreted through bio tensegrity and kinetic-chain concepts. The body functions as an integrated tensegrity system in which bones act as compression elements and myofascial tissues carry tensile forces [54]. Athletes differ in kinetic-chain efficiency, fascial thickness, range of motion, and compensatory movement patterns. Pre-existing fascial densification, fibrosis, or proprioceptive deficits may restrict mechanical energy transfer from proximal to distal segments. Such restrictions could contribute to smaller performance gains irrespective of training sequence [55]. Conversely, favorable fascial elasticity and extracellular matrix glide, whether related to genetic predisposition or previous training, may support faster responses under either sequencing condition [56]. Taken together, the differences in individual CMJ gains may reflect variations in tissue quality, particularly within fascial and tendinous structures, as well as differences in kinetic-chain energy transfer [7]. The larger gains descriptively observed among some athletes who began with PC training remain noteworthy. They suggest that posterior-chain-related structural changes may require time and that earlier exposure could offer a longer period for these changes to develop.

Few studies have directly compared different fascial-chain sequences in relation to CMJ performance. The present study therefore adds to the literature by combining conventional statistical analysis with Neo4j-based relational exploration. The graph structure made it possible to trace how changes were distributed across phases and athletes, even when the group averages were similar. Previous findings on myofascial-chain training provide relevant context. In one study of elite youth football players, two posterior-chain training protocols produced similar CMJ changes, with no significant difference between groups [15]. The authors suggested that the intervention may not have provided a sufficient stimulus to produce meaningful changes in explosive performance. In the present study, the pronounced overall CMJ improvement and wide athlete-level variability suggest that responses may depend on both the selected chain and the magnitude and timing of the mechanical stimulus. Similarly, in-season longitudinal observations in junior team-sport athletes have demonstrated consistent and progressive improvements in explosive strength markers, including countermovement, squat, and drop jumps, under structured training conditions across a competitive season [25]. Another study examined a superficial anterior myofascial-chain strengthening program in youth football players. The intervention increased maximal instep-kicking velocity and produced improvements in muscle activation, myofascial elasticity, and lower-limb kinematics [14]. The reported changes in myofascial elasticity and muscle activation are compatible with the persistent jump improvements observed at Follow-up, particularly among athletes who began with posterior-chain training. Together, these studies indicate that targeting myofascial structures may contribute to explosive performance through elastic energy storage, neuromuscular efficiency, and integrated force transmission. The YBT findings highlight a related but functionally distinct role of the fascial system.

Dynamic balance during the YBT depends on continuous proprioceptive input from mechanoreceptors within deep fascial tissues and joint retinacula. Fascia therefore contributes not only to force transmission but also to sensory function [57, 58]. Research suggests that balance changes following plantar or lower-extremity fascia-oriented interventions may not occur immediately. The central nervous system may require time to integrate new somatosensory information and recalibrate postural control [59]. The Neo4j summaries showed that YBT improvements were not uniform across athletes. Variability was especially high during the Mid-test–Post-test phase. Descriptive sequence-related patterns differed across phases for YBT-R, whereas YBT-L followed a more stable course. The gradual reduction in the rate of improvement may reflect adaptation to new mechanical tension patterns alongside changes in proprioceptive control. Differences between YBT-R and YBT-L, together with heterogeneous early responses, may be associated with functional lower-limb asymmetries and individual differences in fascial stiffness [60]. Dynamic balance may therefore depend on both mechanical and neurosensory processes, which may progress at different rates among athletes. The absence of a significant difference between the second and third YBT-L measurements may also reflect right–left functional asymmetry. Football-specific demands can contribute to side-to-side differences in tissue stiffness, range of motion, and neuromuscular function, reflecting the unilateral and asymmetric nature of many soccer-specific actions [61].

Previous injury may also contribute to altered tissue stiffness and impaired sensory function, potentially affecting force transmission and proprioceptive control [5]. The left lower limb may therefore have differed in baseline elasticity or proprioceptive capacity and responded differently to the intervention. Compensatory mechanisms may also have caused an earlier plateau than on the right side. These explanations remain hypothetical because fascial morphology was not directly assessed. The weaker statistical differences observed in the later YBT phases may reflect a transition from early neural responses to slower changes in fascial and tendinous structures.

The concentration of the largest YBT-R gains within PCFG may be related to the thick tendinous structures of the posterior chain and their role in elastic energy storage through the catapult mechanism [9, 62]. Athlete-level Neo4j analyses nevertheless showed both strong positive responses and limited or negative changes. Balance responses were therefore more heterogeneous than the CMJ responses. This variability is consistent with previous findings showing that individual factors, including training background, fitness level, and sport-specific demands, can influence functional movement and performance relationships in young football players [63].

Densification of hyaluronic acid and fibrosis within the extracellular matrix may also impair fascial force transmission [54]. YBT-L improvements were more evenly distributed, although the lowest values occurred more frequently in SCFG. This pattern may reflect the slower time course of structural change. Spiral-chain training may improve early neural coordination but provide insufficient stimulus for the collagen synthesis required for sustained force production and elastic recoil in fascial and tendinous tissues [16]. This limitation may be particularly relevant for the left lower limb, which often serves a stabilizing role.

As with CMJ, the heterogeneous YBT responses and the absence of a uniform sequence effect may be interpreted within a bio tensegrity framework [54, 64]. Each athlete has a distinct kinetic-chain profile shaped by previous injuries, postural compensations, and fibroblast activity. Cellular and mechanical responses to the same training stimulus may therefore differ substantially among individuals.

The primary inferential analyses demonstrated significant improvements over time in CMJ, YBT-R, and YBT-L but did not establish the overall superiority of either training sequence. The mixed-design ANOVA showed no significant Time × Group interaction for any outcome. The secondary LMM identified a significant Group × Time interaction only for YBT-R. Because this finding was outcome-specific and was not supported for CMJ or YBT-L, it should not be generalized as evidence that one sequence was generally superior.

The KG-derived summaries identified numerical differences in the distribution of performance changes across phases and athletes. For example, different sequences showed numerically larger CMJ changes during different temporal transitions. However, these summaries were obtained from the same observations evaluated in the inferential analyses and did not independently estimate statistical uncertainty. Consequently, the KG findings should be interpreted as exploratory and hypothesis-generating rather than as confirmatory evidence of sequence effects.

The choice of a knowledge graph was based on its compatibility with the relational and longitudinal structure of the dataset and the study’s exploratory query requirements. By representing athletes, training sequences, intervention phases, measurement time points, and performance metrics within a unified structure, the KG facilitated the traceability of athlete-level profiles and the retrieval of phase-specific descriptive summaries. However, the present study did not formally compare KG modeling with conventional statistical, relational database, or other data-management approaches in terms of statistical power, predictive accuracy, model fit, analytical accuracy, query speed, scalability, usability, error reduction, or computational efficiency. Therefore, the findings do not establish the KG as the most appropriate or a superior method for athlete monitoring. Rather, its contribution in the present study lies in complementary relational data organization, longitudinal traceability, and exploratory retrieval of phase- and athlete-specific observations.

## Conclusion

This study found that myofascial chain-oriented training was associated with improvements in CMJ and YBT performance over time in youth football players. No statistically significant overall superiority of either training sequence was demonstrated. The knowledge graph identified numerically different patterns of performance change across intervention phases, but these findings were descriptive and hypothesis-generating rather than inferential evidence of sequence-specific effects. The physiological mechanisms underlying these patterns cannot be established because tissue properties, neuromuscular activation, and related mechanistic variables were not directly measured.

The knowledge graph provided a complementary relational representation of the longitudinal data and enabled exploratory examination of phase-related changes and athlete-level response variability. Future studies should test such patterns using pre specified statistical comparisons and direct physiological measurements.

Accordingly, the present findings suggest that performance adaptations following myofascial chain-oriented training develop over time and may vary between athletes, rather than representing uniform responses across individuals. From a practical perspective, considering the temporal and athlete-specific patterns observed with different myofascial chain sequences may be relevant when designing training interventions, although the present findings do not establish an optimal sequence. The observed improvements over time support further investigation of myofascial chain-oriented training as a performance intervention, while the potential role of sequencing remains uncertain.

Beyond its practical implications, this study provides a methodological contribution to sports science by introducing a knowledge graph-based analytical framework capable of integrating time-dependent changes, chain-sequence patterns, and individual response variability within a unified systems-level model. By moving beyond purely outcome-oriented evaluation and incorporating process-oriented performance analysis, the proposed framework offers a novel perspective for understanding complex athletic adaptations and may contribute to the future development of multidimensional performance modeling approaches in sports science.

Accordingly, the present results support further investigation of myofascial chain-oriented training but do not justify recommending one training sequence over the other. Larger studies incorporating prespecified phase-specific statistical comparisons, direct assessments of fascial tissue properties, and formal evaluation of KG-based analytical frameworks are required before sequence-specific training recommendations can be made.

Several limitations should be considered when interpreting the findings of the present study. First, the research was conducted exclusively on elite adolescent male football players. The mechanical properties, thickness, and training responsiveness of fascial tissues exhibit substantial inter-individual variability depending on factors such as age, sex-related hormonal fluctuations, training background, and body mass index. The sample size was relatively small, which may have limited the statistical power to detect significant interaction effects. Therefore, the findings cannot be directly generalized to female athletes, different age groups (e.g., children or older adults), or amateur and sedentary populations. Exercise progression was applied uniformly by training week rather than individualized to athlete-specific readiness, and formal intensity monitoring (e.g., RPE or %MVC) was not implemented. Testing sessions were standardized for time of day and testing surface, but pre-test fatigue and nutritional status were not systematically monitored or controlled, which may have introduced additional variability into performance measurements across time points. Additionally, the outcome assessor who performed the physical performance assessments was not blinded to the participants’ training sequence assignments due to logistical limitations. Although strictly standardized testing protocols and objective, automated instruments (such as the photocell jump-system) were utilized to minimize potential evaluator bias, the lack of assessor blinding represents a methodological limitation that should be considered when interpreting the results.

In addition, the baseline tissue quality of the athletes could not be controlled or directly assessed. Future studies should support performance measurements with tissue imaging techniques such as high-resolution ultrasonography and elastography, include athletes from different sexes and sporting disciplines, and investigate fascial thickness and glide dynamics in vivo. Such approaches may provide a clearer understanding of the role of fascial training within sports science.

The knowledge graph analysis also has methodological limitations. The KG-derived phase changes and athlete rankings were calculated from the same observations evaluated in the conventional statistical analyses and therefore do not constitute independent confirmatory evidence. The KG queries did not independently estimate statistical uncertainty, p-values, confidence intervals, or effect sizes. In addition, the KG was not formally benchmarked against conventional statistical or tabular workflows in terms of statistical power, predictive accuracy, model fit, computational efficiency, or error reduction. Its added value in the present study is therefore limited to relational data integration, traceability, and exploratory querying rather than inferential or statistical superiority.

## Supplementary Information

Supplementary Material 1.

Supplementary Material 2.

Supplementary Material 3.

Supplementary Material 4.

Supplementary Material 5.

## Data Availability

The datasets used and/or analyzed during the current study are available from the corresponding author on reasonable request, as the raw data are not publicly available to preserve individuals’ privacy under the European General Data Protection Regulation.The original participant-level dataset and populated Neo4j database are not publicly available. The complete computational materials are available in the supplementary material, including the Neo4j graph schema, constraint and index definitions, data-import templates, preprocessing scripts, Cypher queries and step-by-step execution instructions.
