# Supplementary material for: Myofascial chain sequencing and performance adaptations in youth football players: an exploratory knowledge graph-supported analysis
Source: BMC Sports Sci Med Rehabil. 2026 Sep 8;18:520. doi: 10.1186/s13102-026-02066-0 (PMC13693028; doi:10.1186/s13102-026-02066-0)
Supplement: Supplementary file 1 [file 13102_2026_2066_MOESM1_ESM.docx]

**Table A.** Posterior Chain 30-Minute Training Session

| **Session** | **Week** | **Exercise Name** | **Duration / Reps** | **Execution & SBL Focus** |
| --- | --- | --- | --- | --- |
| WARM-UP  (6 Minutes) | Weeks 1-6 | 1. Foot Massage with Ball (RELEASE) | 2 Min  (1 min per foot) | Massaging the plantar fascia with a ball to hydrate and release the main anchor point of the SBL. |
|  | Weeks 1-6 | 2. Posterior Stimulation of Ankles Resting on the Foam Roller (FEEL IT) | 2 Min  (10 reps) | Sitting on the foam roller placed between the ankles and calves, applying 3 sec pressure to activate the lower SBL. |
|  | Weeks 1-6 | 3. Roll Down (MOBILITY) | 2 Min  (5-8 reps) | Standing upright, rolling down the spine vertebra by vertebra, feeling the continuous fascial connection of the SBL from feet to head. |
| MAIN PHASE  (20 Minutes)  1-2 Weeks | Weeks 1-2 | 4. Standing Square Against the Wall (STRETCH) | 4 Min  (3x 45 sec) | Stepping back from the wall to form a square shape, providing a static stretch to the hamstrings and spinal erectors. |
|  | Weeks 1-2 | 5. Dynamic Camel (MOBILITY / STRETCH) | 4 Min  (3x 10 reps) | In an inverted "V" position, rhythmically pushing heels to the floor to stretch the entire SBL. |
|  | Weeks 1-2 | 6. Elephant in Motion (STRETCH) | 4 Min  (10 reps per leg) | Bending and straightening the knee while leaning forward, dynamically opening the posterior leg fascia. |
|  | Weeks 1-2 | 7. Three Feet in Motion (MOBILITY) | 4 Min  (5 reps per leg) | On all fours, extending one leg backward/upward to mobilize the hip and posterior leg with pelvic stability. |
|  | Weeks 1-2 | 8. One Leg Deadlift Against the Wall (STRETCH) | 4 Min  (5 reps per leg) | Leaning forward from the hips with one leg straight back against the wall, intensely stretching the lower SBL. |
| MAIN PHASE  (20 Minutes)  3-4 Weeks | Weeks 3-4 | 4. Round Elephant (STRETCH) | 4 Min  (10 reps per leg) | Drawing circles around the front foot with fingertips to stretch the SBL in multiple directions. |
|  | Weeks 3-4 | 5. Elephant on the Plyo Box (STRETCH) | 4 Min  (5 reps per leg) | Performing the Elephant exercise with the front foot elevated to maximize the SBL stretch angle. |
|  | Weeks 3-4 | 6. Stepping Back and Forth (ENERGY / MOBILITY) | 4 Min  (10 reps per leg) | Rhythmic stepping to warm up the posterior fascial lines and increase tissue elasticity. |
|  | Weeks 3-4 | 7. Standing Square Against the Wall (STRETCH) | 4 Min  (3x 45 sec) | Repeating the static stretch to maintain the newly gained range of motion (ROM). |
|  | Weeks 3-4 | 8. Elastic Bounces (ENERGY) | 4 Min  (3x 30 sec) | Rhythmic bouncing on the spot with slightly bent knees, utilizing the slingshot effect of the Achilles tendon and SBL. |
| MAIN PHASE  (20 Minutes)  5-6 Weeks | Weeks 5-6 | 4. Standing Square With Plyo Box (STRETCH) | 4 Min  (3x 45 sec) | Deepening the square stretch by placing hands on a Plyo Box for maximum SBL elongation. |
|  | Weeks 5-6 | 5. Round Elephant (STRETCH) | 4 Min  (10 reps per leg) | Multi-directional SBL preparation repeated from week 3. |
|  | Weeks 5-6 | 6. Elastic Bounces (ENERGY) | 4 Min  (3x 30 sec) | Bouncing routine to develop the kinetic energy storage and release of the fascia. |
|  | Weeks 5-6 | 7. Standing Swing from Low to High (ENERGY) | 4 Min  (10-15 reps) | Swinging the torso down to stretch the SBL, then exploding upward using fascial elastic recoil. |
|  | Weeks 5-6 | 8. Dynamic Lunge Starter (ENERGY) | 4 Min  (5 reps per leg) | Stepping back into a lunge to pre-stretch the Achilles tendon, then explosively thrusting forward into a Stork position. |
| COOL-DOWN  (4 Minutes) | Weeks 1-6 | 9. Rocking on the Sacrum With a Sensory Ball (RELEASE) | 2 Min (Rhythmic rocking) | Lying supine with a sensory ball under the sacrum, rocking rhythmically to initiate parasympathetic relaxation. |
|  | Weeks 1-6 | 10. Back of the Neck Release With a Foam Roller (RELEASE) | 2 Min (Gentle pressure) | Gently massaging the sub occipital muscles (SBL cornerstone) to completely relax the line. |
