# Supplementary material for: Myofascial chain sequencing and performance adaptations in youth football players: an exploratory knowledge graph-supported analysis
Source: BMC Sports Sci Med Rehabil. 2026 Sep 8;18:520. doi: 10.1186/s13102-026-02066-0 (PMC13693028; doi:10.1186/s13102-026-02066-0)
Supplement: Supplementary file 2 [file 13102_2026_2066_MOESM2_ESM.docx]

**Table B.** Spiral Chain 30-Minute Training Session

| **Session** | **Week** | **Exercise Name** | **Duration / Reps** | **Execution & SL Focus** |
| --- | --- | --- | --- | --- |
| WARM-UP (6 Minutes) | Weeks 1-6 | 1. Thoracic Spine Rotations (MOBILITY) | 2 Min  (5 reps per side) | Rotating the rib cage towards the ceiling from an all-fours or side-lying position to open the SL and spinal mobility. |
|  | Weeks 1-6 | 2. Step With Rotation (FEEL IT / MOBILITY) | 2 Min  (10 reps per side) | Stepping forward while rotating the arms and torso in the opposite direction to feel the right-left spiral balance. |
|  | Weeks 1-6 | 3. Shift Squat (MOBILITY) | 2 Min  (5 reps per side) | Shifting the pelvis laterally while rotating the torso in the opposite direction to soften cross-body fascial connections. |
| MAIN PHASE  (20 Minutes)  1-2 Weeks | Weeks 1-2 | 4. Standing Rotation (FEEL IT) | 4 Min  (8 reps per side) | Standing with arms extended, rotating to perceive the right and left double helix of the Spiral Line. |
|  | Weeks 1-2 | 5. Rotating Split Squat (STRETCH) | 4 Min  (5 reps per side) | Descending into a lunge with the hip against the wall, rotating away to increase the cross-body pull of the SL. |
|  | Weeks 1-2 | 6. Supine Corkscrew (STRETCH) | 4 Min  (4 reps per side) | Lying supine, locking one heel between the toes of the other foot, extending the SL through the lower body pull. |
|  | Weeks 1-2 | 7. Three-Legged Windmill (MOBILITY) | 4 Min  (5 reps per side) | On all fours with one leg extended laterally, reaching the opposite arm to the ceiling for a rotational SL stretch. |
|  | Weeks 1-2 | 8. Rolling Beetle (STRETCH / MOBILITY) | 4 Min  (5 reps per side) | From a side-lying position, opening the arm and leg in opposite directions to create cross-rotation. |
| MAIN PHASE (20 Minutes) 3-4 Weeks | Weeks 3-4 | 4. Standing Corkscrew (STRETCH) | 4 Min  (5 reps per side) | Crossing the legs behind one another to create a lateral bend and stretch the SL's elastic capacity. |
|  | Weeks 3-4 | 5. Supine Corkscrew in Action (STRETCH) | 4 Min  (4 reps per side) | Evolving the supine corkscrew by actively reaching the arms overhead to create maximum 3D twisting. |
|  | Weeks 3-4 | 6. Rotating Split Squat (STRETCH) | 4 Min  (5 reps per side) | Repeating the wall rotational split squat to increase tissue glide and ROM. |
|  | Weeks 3-4 | 7. Diagonal Lunge (ENERGY) | 4 Min  (5 reps per side) | Lunging sideways while pulling the arms back like a bow, then springing back using elastic kinetic energy. |
|  | Weeks 3-4 | 8. Boomerang in Sideways Half-Kneeling Position (MOBILITY) | 4 Min  (5 reps per side) | Performing a rotational stretch in a half-kneeling position to open the pelvic and lower extremity SL connections. |
| MAIN PHASE  (20 Minutes) 5-6 Weeks | Weeks 5-6 | 4. Standing Corkscrew (STRETCH) | 4 Min  (5 reps per side) | Repeating the standing cross-body bend to maintain gained ROM. |
|  | Weeks 5-6 | 5. Diagonal Lunge (ENERGY) | 4 Min  (5 reps per side) | Continuing the boomerang-effect lunge for kinetic energy storage. |
|  | Weeks 5-6 | 6. Dynamic Little Mermaid (ENERGY) | 4 Min  (3 reps per side) | From a "Z" sit, explosively lifting the hips and stretching the torso around the supporting arm using the slingshot effect. |
|  | Weeks 5-6 | 7. Twisting Scorpion (STRETCH) | 4 Min  (5 reps per side) | Prone, bending one leg and reaching it across the body like a scorpion tail to stretch rotational SL connections. |
|  | Weeks 5-6 | 8. Aircraft Marshalling with Clubs (ENERGY) | 4 Min (10 reps) | Crossing and swinging the arms like an aircraft marshaller to integrate the SL with the Arm Lines' elastic recoil. |
| COOL-DOWN  (4 Minutes) | Weeks 1-6 | 9. Prone Corkscrew (RELEASE / STRETCH) | 2 Min  (3 reps per side) | Lying prone, crossing the arms and legs in opposite directions for a deep, static SL release. |
|  | Weeks 1-6 | 10. Balanced Breathing (RELEASE) | 2 Min (Steady breathing) | Supine, focusing on rib cage mobility and expanding the diaphragm to complete the transition to the parasympathetic system. |
