# Supplementary material for: Myofascial chain sequencing and performance adaptations in youth football players: an exploratory knowledge graph-supported analysis
Source: BMC Sports Sci Med Rehabil. 2026 Sep 8;18:520. doi: 10.1186/s13102-026-02066-0 (PMC13693028; doi:10.1186/s13102-026-02066-0)
Supplement: Supplementary file 3 [file 13102_2026_2066_MOESM3_ESM.docx]

| Section/topic | No | CONSORT 2025 checklist item description | Reported on page no. |
| --- | --- | --- | --- |
| **Title and abstract** | | |  |
| Title and structured abstract | 1a | Identification as a randomised trial | Title and Methods (Research design)p:7-8 |
|  | 1b | Structured summary of the trial design, methods, results, and conclusions | Abstract p:1 |
| **Open science** | | |  |
| Trial registration | 2 | Name of trial registry, identifying number (with URL) and date of registration | Abstract (Trial registration) and Declarations p:1 |
| Protocol and statistical analysis plan | 3 | Where the trial protocol and statistical analysis plan can be accessed | Methods and Additional file 1 & 2 p:6-14 |
| Data sharing | 4 | Where and how the individual de-identified participant data (including data dictionary), statistical code and any other materials can be accessed | Declarations (Availability of data and materials) p:38 |
| Funding and conflicts of interest | 5a | Sources of funding and other support (eg, supply of drugs), and role of funders in the design, conduct, analysis and reporting of the trial | Declarations (Funding) and Acknowledgments p:37-38 |
|  | 5b | Financial and other conflicts of interest of the manuscript authors | Declarations (Competing interests) p:38 |
| **Introduction** | | |  |
| Background and rationale | 6 | Scientific background and rationale | Background p:3 |
| Objectives | 7 | Specific objectives related to benefits and harms | Background (Last paragraph before Hypotheses) p:4 |
| **Methods** | | |  |
| Patient and public involvement | 8 | Details of patient or public involvement in the design, conduct and reporting of the trial | Methods (Participants) p:8 |
| Trial design | 9 | Description of trial design including type of trial (eg, parallel group, crossover), allocation ratio, and framework (eg, superiority, equivalence, non-inferiority, exploratory) | Methods (Research design) p:8 |
| Changes to trial protocol | 10 | Important changes to the trial after it commenced including any outcomes or analyses that were not prespecified, with reason | None |
| Trial setting | 11 | Settings (eg, community, hospital) and locations (eg, countries, sites) where the trial was conducted | Methods and Fig. 1 p:7 |
| Eligibility criteria | 12a | Eligibility criteria for participants | Methods (Participants) (Licensed male football players, U15–U16, no injury) p:8 |
|  | 12b | If applicable, eligibility criteria for sites and for individuals delivering the interventions (eg, surgeons, physiotherapists) | Methods (Data collection instruments and protocols) p:9-10-11 |
| Intervention and comparator | 13 | Intervention and comparator with sufficient details to allow replication. If relevant, where additional materials describing the intervention and comparator (eg, intervention manual) can be accessed | Methods (Training program) and Additional file 1 & 2 p:11 |
| Outcomes | 14 | Prespecified primary and secondary outcomes, including the specific measurement variable (eg, systolic blood pressure), analysis metric (eg, change from baseline, final value, time to event), method of aggregation (eg, median, proportion), and time point for each outcome | Methods (countermovement jump& Balance performance) (CMJ, YBT-R, YBT-L, Composite Score formula, measured at 4 time points) p:9-10 |
| Harms | 15 | How harms were defined and assessed (eg, systematically, non-systematically) | Methods (Assessed through standard screening, only healthy participants included) p:8 |
| Sample size | 16a | How sample size was determined, including all assumptions supporting the sample size calculation | Methods (Participants) (G*Power 3.1 analysis details, f=0.25, power=0.95, alpha=0.05) p:8 |
|  | 16b | Explanation of any interim analyses and stopping guidelines | Not applicable (No interim analyses or stopping guidelines were planned) |
| Randomisation: |  |  |  |
| Sequence generation | 17a | Who generated the random allocation sequence and the method used | Methods (Participants) (Randomly assigned in a 1:1 ratio) p:8 |
|  | 17b | Type of randomisation and details of any restriction (eg, stratification, blocking and block size) | Methods (Participants) (Simple 1:1 randomisation into two equal groups) p:8 |
|  |  |  | **Reported on page no.** |
| Allocation concealment mechanism | 18 | Mechanism used to implement the random allocation sequence (eg, central computer/telephone; sequentially numbered, opaque, sealed containers), describing any steps to conceal the sequence until interventions were assigned | Methods (Participants) (Allocation was performed following the completion of pre-test assessments) p:8 |
| Implementation | 19 | Whether the personnel who enrolled and those who assigned participants to the interventions had access to the random allocation sequence | Methods (Participants) p:8 |
| Blinding | 20a | Who was blinded after assignment to interventions (eg, participants, care providers, outcome assessors, data analysts) | Not blinded (Blinding of athletes/coaches was not possible due to the nature of training exercises) |
|  | 20b | If blinded, how blinding was achieved and description of the similarity of interventions | Not applicable |
| Statistical methods | 21a | Statistical methods used to compare groups for primary and secondary outcomes, including harms | Methods (Statistical analysis) (2 × 4 Mixed-Design ANOVA, Mauchly's test, Bonferroni adjustments) p:11-12 |
|  | 21b | Definition of who is included in each analysis (eg, all randomised participants), and in which group | Methods (Statistical analysis) (All 40 randomised participants included, normality tested via Shapiro–Wilk) p:11-12 |
|  | 21c | How missing data were handled in the analysis | Methods (Statistical analysis) and Fig. 1 (Data Processing) (Outlier and missing data control) p:11-12 |
|  | 21d | Methods for any additional analyses (eg, subgroup and sensitivity analyses), distinguishing prespecified from post hoc | Methods (Construction of the knowledge graph-based data model) (Neo4j and Cypher query relational network analysis) p:13-14-15 |
| **Results** | | |  |
| Participant flow, including flow diagram | 22a | For each group, the numbers of participants who were randomly assigned, received intended intervention, and were analysed for the primary outcome | Methods (Participants) and Fig. 1 (Step 1 & 2) (40 participants assigned, all completed the 12-week program) p:8 |
|  | 22b | For each group, losses and exclusions after randomisation, together with reasons | Methods (Participants) and Fig. 1 (No losses or exclusions occurred, n=40 analyzed) p:8 |
| Recruitment | 23a | Dates defining the periods of recruitment and follow-up for outcomes of benefits and harms | Methods (Research design) (Ethical approval date: 28.04.2025, followed by 12-week training and 4-week follow-up)p:8 |
|  | 23b | If relevant, why the trial ended or was stopped | Not applicable (The trial was completed successfully as scheduled) |
| Intervention and comparator delivery | 24a | Intervention and comparator as they were actually administered (eg, where appropriate, who delivered the intervention/comparator, how participants adhered, whether they were delivered as intended (fidelity)) | Methods (Training program) (30-minute sessions, structured warm-up, main section, and cool-down) p:11 |
|  | 24b | Concomitant care received during the trial for each group | Methods (Training program) (Both groups continued their routine club training 2 days per week) p:11 |
| Baseline data | 25 | A table showing baseline demographic and clinical characteristics for each group | Results and Table 1 (Pre-test rows) (Height, weight, and baseline performance scores) p:15-16 |
| Numbers analysed,  outcomes and estimation | 26 | For each primary and secondary outcome, by group:  ● the number of participants included in the analysis  ● the number of participants with available data at the outcome time point  ● result for each group, and the estimated effect size and its precision (such as 95% confidence interval)  ● for binary outcomes, presentation of both absolute and relative effect size | Results , Table 1 , Table 2 , Table 3 , Table 4 (Mean±SD, F-statistics, p-values, partial eta squared, and phase-based mean deltas) p:17-20 |
| Harms | 27 | All harms or unintended events in each group | Results (No sports injuries or negative physical events were reported during the intervention periods) |
| Ancillary analyses | 28 | Any other analyses performed, including subgroup and sensitivity analyses, distinguishing pre-specified from post hoc | Results (Findings obtained through the knowledge graph analysis) , Table 5 , Table 6 , Table 7 , Table 8 , Table 9 (Individual athlete total gains and left/right symmetry evaluations) p:23-24-25 |
| **Discussion** | | |  |
| Interpretation | 29 | Interpretation consistent with results, balancing benefits and harms, and considering other relevant evidence | Discussion p:27-34 |
| Limitations | 30 | Trial limitations, addressing sources of potential bias, imprecision, generalisability, and, if relevant, multiplicity of analyses | Discussion (Last paragraphs focusing on age, sex constraints, and lack of tissue imaging tools) p:33-34 |

Citation: Hopewell S, Chan AW, Collins GS, Hróbjartsson A, Moher D, Schulz KF, et al. CONSORT 2025 Statement: updated guideline for reporting randomised trials. BMJ. 2025; 388:e081123. <https://dx.doi.org/10.1136/bmj-2024-081123>
© 2025 Hopewell et al. This is an Open Access article distributed under the terms of the Creative Commons Attribution License (<https://creativecommons.org/licenses/by/4.0/>), which permits unrestricted use, distribution, and reproduction in any medium, provided the original work is properly cited.

*We strongly recommend reading this statement in conjunction with the CONSORT 2025 Explanation and Elaboration and/or the CONSORT 2025 Expanded Checklist for important clarifications on all the items. We also recommend reading relevant CONSORT extensions. See [www.consort-spirit.org](http://www.consort-spirit.org).
