# Supplementary material for: Myofascial chain sequencing and performance adaptations in youth football players: an exploratory knowledge graph-supported analysis
Source: BMC Sports Sci Med Rehabil. 2026 Sep 8;18:520. doi: 10.1186/s13102-026-02066-0 (PMC13693028; doi:10.1186/s13102-026-02066-0)
Supplement: Supplementary file 5 [file 13102_2026_2066_MOESM4_ESM.zip › Supplementary material /Supplementary Table S1, S2, and S3.docx]

**Supplementary Tables**

Table S1

Individual CMJ total gains of the five athletes with the highest and lowest changes from Pre-test to Follow-up

| Athlete | Chain Sequence | Total Gain: Follow-up − Pre-test (cm) |
| --- | --- | --- |
| 23 | SC🡪PC | 19.81 |
| 16 | PC🡪SC | 18.99 |
| 31 | SC🡪PC | 15.04 |
| 21 | PC🡪SC | 15.01 |
| 11 | PC🡪SC | 12.12 |
| ... | ... | ... |
| 3 | PC🡪SC | 2.45 |
| 7 | PC🡪SC | 2.40 |
| 37 | SC🡪PC | 1.71 |
| 6 | SC🡪PC | 1.51 |
| 38 | SC🡪PC | 0.99 |

Athlete numbers are pseudonymous participant identifiers. Total Gain was calculated for each athlete as Follow-up CMJ minus Pre-test CMJ and is expressed in centimeters. Positive values indicate improvement, whereas negative values indicate a decrease. PC→SC = posterior-chain training during the first intervention phase followed by spiral-chain training during the second phase; SC→PC = spiral-chain training during the first phase followed by posterior-chain training during the second phase; CMJ = countermovement jump.

Table S2

Individual YBT-R total gains of the five athletes with the highest and lowest changes from Pre-test to Follow-up

| Athlete | Chain Sequence | Total Gain: Follow-up − Pre-test (percentage points) |
| --- | --- | --- |
| 22 | PC🡪SC | 35.9921 |
| 27 | PC🡪SC | 29.4683 |
| 29 | SC🡪PC | 21.7086 |
| 15 | SC🡪PC | 21.4244 |
| 11 | PC🡪SC | 13.2275 |
| ... | ... | ... |
| 7 | PC🡪SC | -1.8301 |
| 38 | SC🡪PC | -2.9030 |
| 36 | SC🡪PC | -3.4379 |
| 39 | SC🡪PC | -5.0873 |
| 25 | PC🡪SC | -8.2889 |

Athlete numbers are pseudonymous participant identifiers. Total Gain was calculated for each athlete as the normalized Follow-up YBT-R score minus the normalized Pre-test YBT-R score and is expressed in percentage points. Positive values indicate improvement, whereas negative values indicate a decrease. YBT-R = Y-Balance Test score with the right leg as the stance leg; PC→SC = posterior-chain training followed by spiral-chain training; SC→PC = spiral-chain training followed by posterior-chain training; PC = posterior chain; SC = spiral chain.

Table S3

Individual YBT-L total gains of the five athletes with the highest and lowest changes from Pre-test to Follow-up

| Athlete | Chain Sequence | Total Gain: Follow-up − Pre-test (percentage points) |
| --- | --- | --- |
| 27 | PC🡪SC | 29.5249 |
| 29 | SC🡪PC | 23.3978 |
| 13 | SC🡪PC | 20.1401 |
| 22 | PC🡪SC | 19.1358 |
| 18 | PC🡪SC | 10.2290 |
| ... | ... |  |
| 7 | PC🡪SC | -0.4673 |
| 25 | PC🡪SC | -0.8603 |
| 10 | PC🡪SC | -1.2346 |
| 39 | SC🡪PC | -8.3652 |
| 38 | SC🡪PC | -8.7824 |

Athlete numbers are pseudonymous participant identifiers. Total Gain was calculated for each athlete as the normalized Follow-up YBT-L score minus the normalized Pre-test YBT-L score and is expressed in percentage points. Positive values indicate improvement, whereas negative values indicate a decrease. YBT-L = Y-Balance Test score with the left leg as the stance leg; PC→SC = posterior-chain training followed by spiral-chain training; SC→PC = spiral-chain training followed by posterior-chain training; PC = posterior chain; SC = spiral chain.
