# Supplementary material for: Myofascial chain sequencing and performance adaptations in youth football players: an exploratory knowledge graph-supported analysis
Source: BMC Sports Sci Med Rehabil. 2026 Sep 8;18:520. doi: 10.1186/s13102-026-02066-0 (PMC13693028; doi:10.1186/s13102-026-02066-0)
Supplement: Supplementary file 5 [file 13102_2026_2066_MOESM4_ESM.zip › Supplementary material /Neo4J Supplementary Materials/Queries.pdf]

For T0-T1 pass (Table 5 Column 1 mean delta):

```
"MATCH (a:Athlete)-[:HAS_OBSERVATION]->(o0:Observation)-
[:OF_METRIC]->(:Metric {name:"CMJ"}),
(o0)-[:AT_TIME]->(:Timepoint {time:"T0"})
MATCH (a)-[:HAS_OBSERVATION]->(o1:Observation)-[:OF_METRIC]-
>(:Metric {name:"CMJ"}),
(o1)-[:AT_TIME]->(:Timepoint {time:"T1"})
WHERE o0.value IS NOT NULL AND o1.value IS NOT NULL
RETURN a.order AS ord, count(*) AS n, avg(o1.value - o0.value)
AS mean_delta
ORDER BY ord;",
```

For T0-T1 pass (Table 5 Column 1 SD delta):

```
"MATCH (a:Athlete)-[:HAS_OBSERVATION]->(o0:Observation)-
[:OF_METRIC]->(:Metric {name:"CMJ"}),
      (o0)-[:AT_TIME]->(:Timepoint {time:"T0"})
MATCH (a)-[:HAS_OBSERVATION]->(o1:Observation)-[:OF_METRIC]-
>(:Metric {name:"CMJ"}),
      (o1)-[:AT_TIME]->(:Timepoint {time:"T1"})
WHERE o0.value IS NOT NULL AND o1.value IS NOT NULL
RETURN a.order AS ord,
       count(*) AS n,
       avg(o1.value - o0.value) AS mean_delta,
       stdevp(o1.value - o0.value) AS sd
ORDER BY ord;"
```

For T1-T2 pass (Table 5 Column 2 mean delta and SD delta):

```
"MATCH (a:Athlete)-[:HAS_OBSERVATION]->(o1:Observation)-
[:OF_METRIC]->(:Metric {name:"CMJ"}),
      (o1)-[:AT_TIME]->(:Timepoint {time:"T1"})
MATCH (a)-[:HAS_OBSERVATION]->(o2:Observation)-[:OF_METRIC]-
>(:Metric {name:"CMJ"}),
      (o2)-[:AT_TIME]->(:Timepoint {time:"T2"})
WHERE o1.value IS NOT NULL AND o2.value IS NOT NULL
RETURN a.order AS ord,
       avg(o2.value - o1.value) AS mean_delta,
       stdevp(o2.value - o1.value) AS sd
ORDER BY ord;"
```

For T2-T3 pass (Table 5 Column 3 mean delta and SD delta):

```
"MATCH (a:Athlete)-[:HAS_OBSERVATION]->(o1:Observation)-
[:OF_METRIC]->(:Metric {name:"CMJ"}),
      (o1)-[:AT_TIME]->(:Timepoint {time:"T2"})
MATCH (a)-[:HAS_OBSERVATION]->(o2:Observation)-[:OF_METRIC]-
>(:Metric {name:"CMJ"}),
      (o2)-[:AT_TIME]->(:Timepoint {time:"T3"})
WHERE o1.value IS NOT NULL AND o2.value IS NOT NULL
RETURN a.order AS ord,
       avg(o2.value - o1.value) AS mean_delta,
```

```
       stdevp(o2.value - o1.value) AS sd
ORDER BY ord;"
```

For T1-T3 pass (Table 5 Column 4 mean total gain and SD):

```
"MATCH (a:Athlete)-[:HAS_OBSERVATION]->(o0:Observation)-
[:OF_METRIC]->(:Metric {name:"CMJ"}),
      (o0)-[:AT_TIME]->(:Timepoint {time:"T0"})
MATCH (a)-[:HAS_OBSERVATION]->(o3:Observation)-[:OF_METRIC]-
>(:Metric {name:"CMJ"}),
      (o3)-[:AT_TIME]->(:Timepoint {time:"T3"})
WHERE o0.value IS NOT NULL AND o3.value IS NOT NULL
RETURN a.order AS ord,
       avg(o3.value - o0.value) AS total_gain,
       stdevp(o3.value - o0.value) AS sd
ORDER BY ord;"
```

For Table 6:

```
MATCH (a:Athlete)

MATCH (a)-[:HAS_OBSERVATION]->(o0:Observation)-[:OF_METRIC]-
>(:Metric {name:"YBalanceComposite"}),
      (o0)-[:AT_TIME]->(:Timepoint {time:"T0"})
WHERE o0.side = "Right"

MATCH (a)-[:HAS_OBSERVATION]->(o1:Observation)-[:OF_METRIC]-
>(:Metric {name:"YBalanceComposite"}),
      (o1)-[:AT_TIME]->(:Timepoint {time:"T1"})
WHERE o1.side = "Right"

MATCH (a)-[:HAS_OBSERVATION]->(o2:Observation)-[:OF_METRIC]-
>(:Metric {name:"YBalanceComposite"}),
      (o2)-[:AT_TIME]->(:Timepoint {time:"T2"})
WHERE o2.side = "Right"

MATCH (a)-[:HAS_OBSERVATION]->(o3:Observation)-[:OF_METRIC]-
>(:Metric {name:"YBalanceComposite"}),
      (o3)-[:AT_TIME]->(:Timepoint {time:"T3"})
WHERE o3.side = "Right"

WITH
a.order AS ord,
toFloat(replace(o0.value,",",".")) AS t0,
toFloat(replace(o1.value,",",".")) AS t1,
toFloat(replace(o2.value,",",".")) AS t2,
toFloat(replace(o3.value,",",".")) AS t3

UNWIND [
  {phaseName:"T0-T1", delta:t1-t0, ord:ord},
  {phaseName:"T1-T2", delta:t2-t1, ord:ord},
  {phaseName:"T2-T3", delta:t3-t2, ord:ord}
] AS x

RETURN
x.phaseName AS phaseName,
x.ord AS ord,
count(*) AS n,
round(avg(x.delta)*10000)/10000.0 AS mean_delta,
round(stDev(x.delta)*10000)/10000.0 AS sd_delta,
round(percentileCont(x.delta,0.5)*10000)/10000.0 AS
median_delta
ORDER BY
phaseName,
ord;
```

For Table 7:

```
MATCH (a:Athlete)

MATCH (a)-[:HAS_OBSERVATION]->(o0:Observation)-[:OF_METRIC]-
>(:Metric {name:"YBalanceComposite"}),
      (o0)-[:AT_TIME]->(:Timepoint {time:"T0"})
WHERE o0.side = "Left"

MATCH (a)-[:HAS_OBSERVATION]->(o1:Observation)-[:OF_METRIC]-
>(:Metric {name:"YBalanceComposite"}),
      (o1)-[:AT_TIME]->(:Timepoint {time:"T1"})
WHERE o1.side = "Left"

MATCH (a)-[:HAS_OBSERVATION]->(o2:Observation)-[:OF_METRIC]-
>(:Metric {name:"YBalanceComposite"}),
      (o2)-[:AT_TIME]->(:Timepoint {time:"T2"})
WHERE o2.side = "Left"

MATCH (a)-[:HAS_OBSERVATION]->(o3:Observation)-[:OF_METRIC]-
>(:Metric {name:"YBalanceComposite"}),
      (o3)-[:AT_TIME]->(:Timepoint {time:"T3"})
WHERE o3.side = "Left"

WITH
    a.order AS ord,
    toFloat(replace(o0.value,",",".")) AS t0,
    toFloat(replace(o1.value,",",".")) AS t1,
    toFloat(replace(o2.value,",",".")) AS t2,
    toFloat(replace(o3.value,",",".")) AS t3

UNWIND [
    {phaseName:"T0–T1", delta:t1-t0, ord:ord},
    {phaseName:"T1–T2", delta:t2-t1, ord:ord},
    {phaseName:"T2–T3", delta:t3-t2, ord:ord}
] AS x

RETURN
    x.phaseName AS phaseName,
    x.ord AS ord,
    count(*) AS n,
    round(avg(x.delta)*10000)/10000.0 AS mean_delta,
    round(stDev(x.delta)*10000)/10000.0 AS sd_delta,
    round(percentileCont(x.delta,0.5)*10000)/10000.0 AS
median_delta

ORDER BY
    phaseName,
    ord;
```
